# Two-Dimensional Mapping of Arsenic Concentration and Speciation with Diffusive Equilibrium in Thin-Film Gels

**DOI:** 10.1021/acs.est.3c00887

**Published:** 2023-05-16

**Authors:** Andrea Castillejos Sepúlveda, Edouard Metzger, Sten Littmann, Heidi Taubner, Arjun Chennu, Lais Gatti, Dirk de Beer, Judith M. Klatt

**Affiliations:** † Microsensor Group, Max Planck Institute for Marine Microbiology, Celsiusstraße 1, Bremen 28359, Germany; ‡ Laboratoire de Planétologie et Géosciences, Université d’Angers, Nantes Université, Le Mans Université, CNRS UMR 6112, Angers 49045, France; § Biogeochemistry Group, Max Planck Institute for Marine Microbiology, Celsiusstraße 1, Bremen 28359, Germany; ∥ MARUM Center for Marine Environmental Science and Faculty of Geosciences, Organic Geochemistry Group, University of Bremen, Leobener Str. 8, Bremen 28359, Germany; ⊥ Data Science and Technology, Leibniz Centre for Tropical Marine Research, Fahrenheitstr. 6, Bremen 28359, Germany; # Microcosm Earth Center, Max Planck Institute for Terrestrial Microbiology and Philipps-Universität Marburg, Marburg 35032, Germany; ∇ Center for Synthetic Microbiology (SYNMIKRO), Marburg 35032, Germany; ○ Biogeochemistry Group, Department of Chemistry, Philipps-Universität Marburg, Marburg 35032, Germany

**Keywords:** arsenate, arsenite, phosphate, probe, element mapping, redox reaction

## Abstract

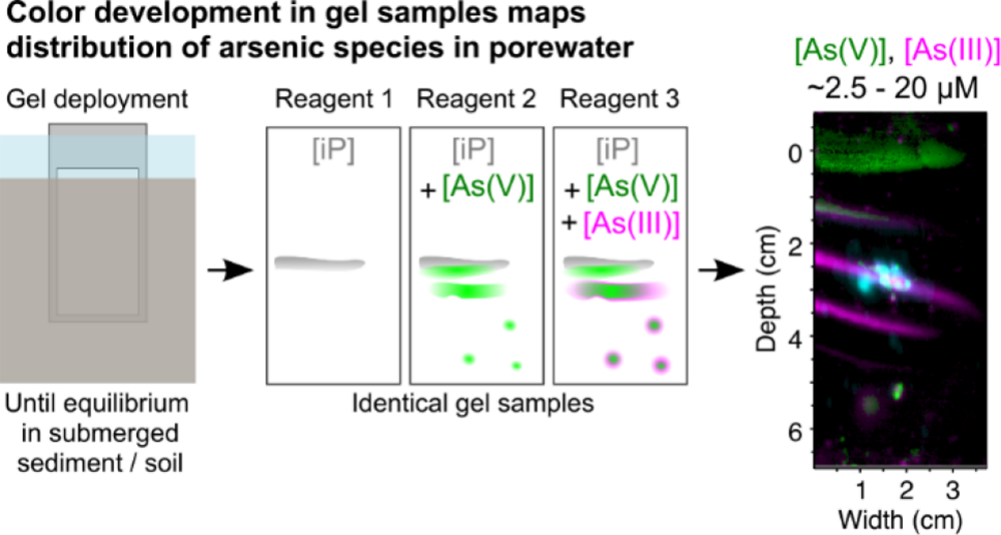

We present a new approach combining diffusive equilibrium in thin-film gels and spectrophotometric methods to determine the spatial distribution of arsenite, arsenate, and phosphate at submillimeter resolution. The method relies on the simultaneous deployment of three gel probes. Each retrieved gel is exposed to malachite green reagent gels differing in acidity and oxidant addition, leading to green coloration dependent on analyte speciation and concentration. Hyperspectral images of the gels enable mapping the three analytes in the 2.5–20 μM range. This method was applied in a contaminated brook in the Harz mountains, Germany, together with established mapping of dissolved iron. The use of two-dimensional (2D) gel probes was compared to traditional porewater extraction. The gels revealed banded porewater patterns on a mm-scale, which were undetectable using traditional methods. Small-scale correlation analyses of arsenic and iron microstructures in the gels suggested active iron-driven local redox cycling of arsenic. Overall, the results indicate continuous net release of arsenic from contaminant particles and deepen our understanding of arsenate transformation under anaerobic conditions. This study is the first fine-scale 2D characterization of arsenic speciation in porewater and represents a crucial step toward understanding the transfer and redox cycling of arsenic in contaminated sediment/soil ecosystems.

## Abstract 2

A novel method generates images of porewater arsenic speciation to assess fine-scale redox cycling in contaminated soil and sediment, which is crucial to understanding arsenic mobility in the environment.

## 1 Introduction

The urgent need to limit human exposure to arsenic, a naturally occurring carcinogen, drives the study of arsenic mobility in ecosystems connected to the food web, such as rice paddies irrigated by groundwater contaminated by arsenic, mostly in inorganic form.^1^ Our understanding of arsenic cycling in soils and sediment relies on porewater measurements obtained either by rhizone sampling in sediment cores, squeezing, or centrifuging a slice of sediment, yielding depth resolution in the cm scale. However, smaller-scale environmental or biological factors often drive biogeochemical spatio-temporal dynamics. Depth gradients from extracted porewater reflect only the average concentration and speciation of an analyte, obscure “hotspots”, and cannot adequately account for horizontal heterogeneity. Thus, the microenvironmental processes shaping arsenic speciation, and hence mobility, may be obscured.^2,3^ These limitations highlight the need for fine-scale two-dimensional (2D) imaging of arsenic speciation in soils and sediments.

Several techniques have been developed to generate analyte maps in 2D, such as peepers, “Diffusive equilibrium in thin-films” (DET), and “Diffusive Gradients in thin-films” (DGT). All three of these techniques can be applied in either soils or sediments, as long as these are fully submerged, due to their dependence on diffusion of analytes from porewater. Compared to peepers, DETs generate higher spatial and temporal resolution images and can characterize porewater solutes in heterogeneous environments. Solute distribution is determined by placing a sampling gel in soil or sediment until equilibrium with analytes in the porewater is reached. Average concentration of analytes over an area can be obtained by subsequent analysis of gel slices, yielding mm resolution in 2D.^4,5^ DGTs differ fundamentally from DETs by measuring analyte fluxes through the active removal of analyte from the environment.^6^ This leads to lower detection limits due to analyte accumulation. However, models are required to back-calculate the environmental concentration of an analyte from DGT samples, and active removal may impact processes during deployment.^7^ While DGTs are therefore useful in assessing fluxes, DETs directly mirror the environmental analyte concentration at equilibrium and may, however, underestimate the actual porewater concentration.

Furthermore, the measurement of arsenic concentration in DET gel slices relies on methods which yield either only total arsenic concentration^8^ or depend on highly specialized equipment like ICP-MS^9^ or a synchrotron.^10,11^ Additionally, improper sample storage may change arsenic speciation.^12^ Colorimetric assays represent a quick, cost-efficient alternative to analyze porewater or gel slices.^13−15^ For other analytes, colorimetry has even be applied directly on intact gels, omitting the slicing step.^16−19^ Briefly, retrieved gels are reacted with colorimetric reagents and then scanned with a scanner or hyperspectral camera to assess local analyte concentration. Among others, colorimetric methods used with gel probes include bromo-phenol blue for alkalinity,^20−22^ Griess for nitrite and nitrate,^19^ ferrozine for iron,^16,23^ and molybdenum blue for phosphate.^16,24^

Since phosphate and arsenate have similar properties, these molecules often compete for binding sites in analytical reagents,^25^ proteins,^26^ and minerals.^27^ Thus, some measure of interference is expected in methods targeting phosphate or As(V). Taking advantage of this property, the molybdenum blue method was adapted for As(V) and As(III) measurement in cuvettes.^15^ However, fine-scale arsenic speciation measurements in 2D using DETs were not previously reported.

The most crucial aspect of adapting a colorimetric assay for application in DETs is consideration of the tradeoffs between sensitivity and equilibrium time, as well as reaction time and 2D resolution. Colorimetric methods requiring long reaction times result in a loss of 2D resolution by allowing analyte diffusion within the sample gel before complexation. Quick-acting colorimetric reagents forming large, slowly diffusing, complexes favor the preservation of 2D structures. For instance, the molybdenum blue method adaptation to gels has been shown to accurately reflect the 2D distribution of phosphate because molybdenum–phosphate complex formation occurs rapidly, and the resulting complex is relatively large.^16^ The recent adaptation of this assay for arsenic speciation exhibits slower color formation because malachite green is used to stain the colorless complex.^15^ Yet, initial complex formation is quick. Also, cross-sensitivity to iron, silicate, and sulfide was already successfully eliminated, overall qualifying this assay as an ideal candidate for arsenic speciation mapping in DETs.

In summary, to target fine-scale arsenic concentration dynamics we aimed to develop a method that (1) differentiates As(V) and As(III) on a micromolar scale, (2) conserves spatial information on a sub-cm scale, (3) is accessible for most labs, (4) is field-usable, and (5) is capable of the same-day data delivery. We therefore adapted an existing colorimetric cuvette method for the 2D mapping of arsenite and arsenate in porewater. To obtain the urgently needed fine-scale information about the distribution of arsenic species, and their spatial correlation with other solutes directly in the environment, severe improvements to the originally described cuvette-based assay were required. To maximize sensitivity and to minimize interference between analytes, as well as the loss of spatial information, we therefore simultaneously optimized spectral measurement parameters and reaction time.

To test the method, we chose the arsenic-contaminated Bossegraben brook in the Harz mountains, Germany, as our study site.^15^ The soil bed of the brook exhibits pronounced spatial heterogeneity of dissolved arsenic species and iron in the porewater. Since the mid-20th century, direct input of iron oxide-sorbed arsenic was greatly decreased.^15^ We hypothesized that previously deposited iron oxides in the soil could still locally leach arsenic, controlled by the local redox cycling of arsenic and iron species. However, because previously collected data on arsenic speciation were based on bulk and porewater measurements, we could not anticipate exactly how the new results obtained through this method would help clarify fine-scale arsenic cycling in the Bossegraben. Through the exploratory first application presented here, we were able to examine previously unknown dynamics between As(V), As(III), inorganic phosphate, and total iron in this site. Beyond applying the novel method for DET gel-based mapping of arsenic speciation over two dimensions at the sub-mm scale, we performed traditional porewater extraction and characterized the solid phase by acquiring high-resolution element maps by micro X-ray fluorescence spectrometry (μXRF).

## 2 Materials and Methods

### 2.1 Principles of Colorimetric Arsenic Speciation Analysis in DETs

To quantify the most common inorganic arsenic species in the environment, arsenite (AsO_3_^3–^, hereafter “As(III)”) and arsenate (AsO_4_^3–^, hereafter “As(V)”), as well as reactive inorganic phosphate (iP), we modified a colorimetric method for porewater analysis^15^ based on the molybdenum blue colorimetric method for iP detection.^28^ Color development, measured as an ‘index of reflectance’ (RI), is based on the formation of a molybdenum complex with either iP or As(V), but not with As(III).^29^ Under highly acidic conditions, color development based on As(V)–Mo complex formation is greatly inhibited.^15,30^ Thus, [iP] and [As(V)] can be distinguished by treating two identical samples with reagents at different acidities: under high acidity color development depends mainly on [iP], under low acidity it depends on both [iP] and [As(V)]. As(III) can be measured after oxidation to As(V). Under low acidity with the addition of oxidant, color development will be dependent on [iP], [As(V)], and surplus [As(V)] resulting from As(III) oxidation. Since each reagent therefore stains a specific set of analytes, concentrations of each analyte can be calculated as a function of color development in all reagents (see Section 2.3). This simple calculation approach can become more complicated at high [As(V)] because a weak coloration response may occur even at high acidities. If [iP] is also high, [As(V)] may be underestimated unless a modified set of equations is used (eqs 4–5 in Section 2.3).

The colorimetric method was previously used to measure the concentration of As(V), As(III), and iP in liquid samples using cuvettes. Two cuvettes were necessary for this procedure: one with high-acidity reagent, targeting mainly phosphate, and the other with low-acidity reagent targeting both arsenate and phosphate. After an initial measurement with a spectrophotometer, KIO_3_ was added to the low-acidity sample to oxidize arsenite to arsenate and to obtain the final measurement encompassing all analytes. A similar procedure, however, could not be followed using DET gels. This is because increasing reaction time for a single sample gel, by treating it first with low-acidity reagent and then with an oxidizing reagent, would allow more time for analytes to diffuse through the sample gel. Consequently, there would be a loss of 2D precision in the final image. To obtain DET images that preserve the spatial information of fine structures, a new protocol with three separate reagents had to be implemented and optimized.

**Figure 1:**
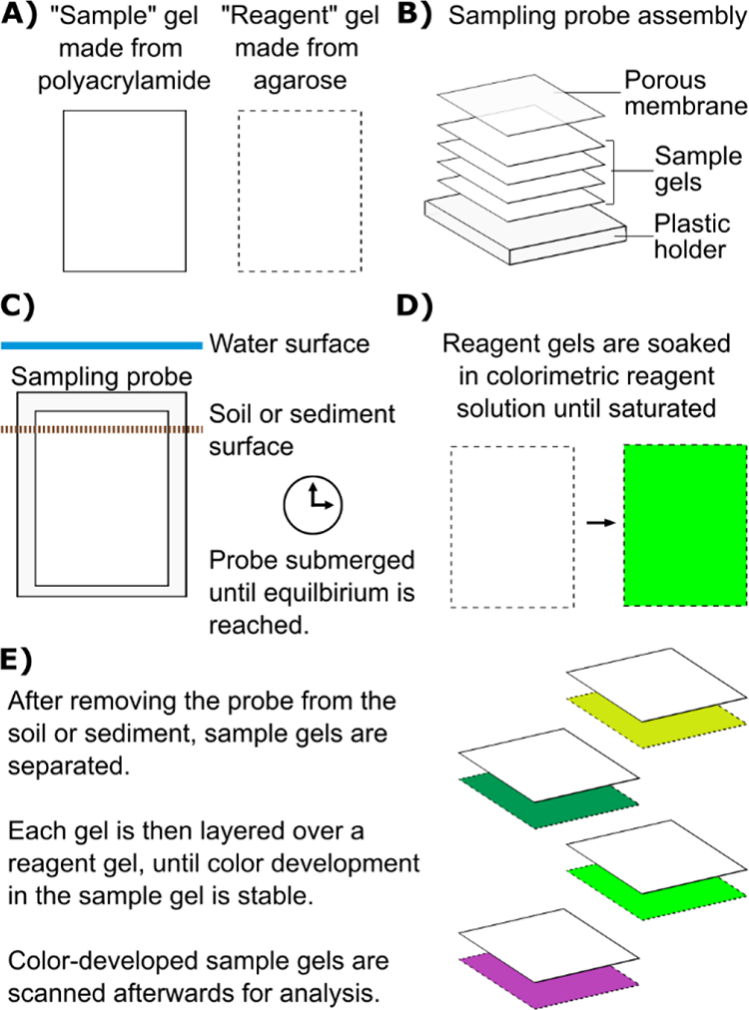
(A–E) Schematic representation of colorimetric analysis of diffusive equilibrium in thin-film (DET) gel probes for application in soil or sediments. Green colors represent the three reagents gels necessary for arsenic and inorganic phosphate determination, while purple represents the reagent gel used for iron determination with the established Ferrozine method.

To adapt the method for DETs, three identical samples were taken using polyacrylamide gels (Figure 1A). These gels were stacked onto a sampling probe or holder (Figure 1B) and inserted into soil until equilibrium was reached between the arsenic and phosphate concentrations in the gels and the surrounding environment (Figure 1C). After retrieval, each gel was placed on an agarose reagent gel (Figure 1E). Each reagent gel was previously soaked in either (1) high acidity, (2) low acidity, or (3) low acidity + oxidant reagent (Figure 1D). After appropriate reaction time, the reagent-based color development, RI, was monitored by hyperspectral imaging.

To optimize the DET method, we (1) minimized reaction time to account for diffusional relaxation (Supp. Methods 1.1), (2) performed calibrations in ultrapure water as well as in artificial saltwater (ASW) to explore interference from sulfate, and (3) optimized measurement parameters, i.e., RI, through spectral analysis of standards and analysis of percentage recovery of each analyte in mixtures of As(V), As(III) and iP. Steps (1) and (3) were closely intertwined as the RI substantially impacts linearity of the dependency on concentration, sensitivity, accuracy, and apparent reaction rate.

The method was applied to a soil core from an arsenic-rich brook in the Harz mountains, Germany. [As(V)], [As(III)], and [iP] were calculated by multivariate analysis on calibrations in standard gels. The established ferrozine colorimetry method was applied to a fourth sample gel. The resulting 2D maps were compared to μXRF imaging, and traditional porewater analysis of arsenic, phosphate, and iron sampled from three additional cores.

### 2.2 DET Preparation

Polyacrylamide gels (hydrated: 0.5 mm thickness) and agarose gels (hydrated: 1 mm thickness; 1.5%, low-melting point agarose) were prepared as previously described^18^ (Supp. Methods 1.2 and Supp. Figure S1).

All chemicals were obtained from Sigma-Aldrich, except H_2_SO_4_, oxalic acid, HCl (Merck), and KIO_3_ (Fisher Scientific).

#### 2.2.1 Malachite Green Reagent

The concentration of reagents used for arsenic and phosphate determination was adapted from the PVA protocol in Castillejos Sepúlveda et al.^15^ by considering that reagent in the reagent gel (thickness: 1 mm) will be diluted by porewater in the analyte gel (thickness: 0.5 mm) by a factor of 2/3, equivalent to the dilution factor in the original protocol. Final reagent solutions (see Section 2.2.2) were prepared fresh for every use.^15^ Malachite green solution and PVA solution can be prepared beforehand. Malachite green solution is a concentrated solution containing the color-developing components, PVA solution contains surfactant necessary to achieve low limits of detection.

##### 2.2.1.1 Malachite Green Solution

A total of 116 mL of 96% H_2_SO_4_ was diluted with ultrapure water to 400 mL of total volume. After cooling to room temperature, 72 g of ammonium molybdate tetrahydrate and 0.36 g of malachite green oxalate salt were added. The solution was diluted to 1 L, stored overnight at 4 °C, and filtered through a 0.2 μm PES filter (Thermo Scientific Rapid-Flow Filter).

##### 2.2.1.2 PVA Solution

A total of 1.33 g poly(vinyl) alcohol were diluted in 1 L of ultrapure water, heated to 80 °C while mixing, and cooled before use.

#### 2.2.2 Reagent Gels

Agarose gels were soaked in 150 mL of one of the following solutions for 3.5 h, in the dark: high-acidity reagent (H) for color development based on iP) 37.5 mL ultrapure water, 18.75 mL of 1% oxalic acid solution, 18.75 mL acetone, 25 mL 6 M H_2_SO_4_, 25 mL PVA solution, and 25 mL malachite green solution.

##### 2.2.2.1 Low-Acidity Reagent (L) for Color Development Based on iP + As(V)

37.5 mL ultrapure water, 18.75 mL of 1% oxalic acid solution, 18.75 mL acetone, 25 mL 2.4 M H_2_SO_4_, 25 mL PVA solution, and 25 mL malachite green solution.

##### 2.2.2.2 Low-Acidity Reagent + Oxidant (LO) for Color Development Based on iP + As(V) + As(III)

18.75 mL ultrapure water, 18.75 mL oxalic acid solution (1%), 18.75 mL acetone, 25 mL 2.4 M H_2_SO_4_, 25 mL PVA solution, 25 mL malachite green solution, and 18.75 mL 10.4 mM KIO_3_.

##### 2.2.2.3 Ferrozine (Adapted from Viollier et al.^23^) for Color Development Based on Fe(II)

2.5 g of ferrozine were dissolved in 250 mL of 0.1 M ammonium acetate. From the resulting solution, 41.1 mL were diluted with ultrapure water to 150 mL.

##### 2.2.2.4 Reducing Gel for Ferrozine (Adapted from Viollier et al.^23^) for the Reduction of Fe(III)

Reducing solution was made by dissolving 25 g of hydroxylamine hydrochloride in 250 mL of 2 M analytical grade HCl. Ammonium acetate buffer (10 M) was made by dissolving 193 g of ammonium acetate in 250 mL of ultrapure water, adjusted to pH 9.5 with ammonium hydroxide. For gel soaking, 61.2 mL of reducing solution and 20.25 mL of ammonium acetate buffer were added to 68.55 mL of ultrapure water.

#### 2.2.3 Calibration Gels

Calibrations were done using gels of the same thickness and material as for sampling. Strips of ∼10 × 4 cm polyacrylamide gels were cut and pressed horizontally between two acrylic blocks, based on the calibration setup from Cesbron et al.^16^ Standards of As(V), As(III), and iP (2 mL each), prepared as previously described,^15^ were pipetted onto holes (2.5 ID) drilled in the upper acrylic block and left to diffuse into the polyacrylamide gel (‘standard gel’) for 1 h. Afterward, standard gels were removed from the blocks and placed over a reagent gel.

This procedure was repeated with three standard gels, one for each reagent mixture (H, L, and LO). Gels were scanned every 15 min for 2.5 h after initial contact between reagent and standard gels.

For iron calibrations, Fe(III) standards in the range of 100–500 μM were prepared according to Viollier et al.^23^ and added to the gels as described above. The standard gel was placed over a ferrozine reagent gel, and the reducing reagent gel was placed over the standard gel. Gels were imaged with a hyperspectral camera immediately after contact with the reducing reagent. Scans were made after 15 min for the ferrozine gels and every 15 min for 2–3 h for the malachite green exposed gels.

### 2.3 Hyperspectral Imaging and Spectral Analysis

Gels were scanned using a Resonon Pika II hyperspectral camera, as previously described.^31^ Scans were used to record radiance images of 0.2 mm per pixel in 462 bands of 1 nm over 400–900 nm. Reflectance images were derived by normalizing the radiance spectra of all pixels to the average radiance of a standard reference board in each image.

In standard gels, regions of interest (ROI) corresponding to known concentrations were manually selected. For iron, RI = reflectance at 562 nm divided by reflectance at 750 nm (*R*_562_/*R*_750_) (adapted from Viollier et al.^23^). For As(V), As(III), and iP, the RI was obtained by calculating integrals, hereafter ‘area under the curve’ (AUC). Overall, around 90 RIs, including differences and ratios, were tested to optimize sensitivity, accuracy, and reaction time. RIs were also calculated from RGB wavelengths (640, 550, 460 nm), to allow the scanning of gels on flatbed scanners. Linear regressions for calibrations of As(V), As(III), iP, and iron in their respective reagents were calculated from mean RI per known analyte concentration.

To determine [As(V)], [As(III)], and [iP] in mixed samples, it was considered that the RI in gels treated with high-acidity reagent (subscript H) only depends on iP,^15,30^ thus
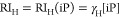
1where γ_H_ is the slope of a regression passing through zero. In gels treated with low acidity reagent (subscript L), RI depends on both iP and As(V) according to
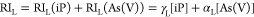
2where α_L_ is the slope of the regression for As(V) standards, and γ_L_ is the slope of iP standards. In sample gels treated with low acidity + oxidant reagent (subscript LO), RI depends on all analytes, since As(III) is oxidized to As(V), according to
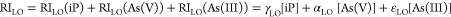
3

Regression parameters, γ, α, and ε, are determined from the calibrations in the three reagents and used to solve eqs 1–3 for all analyte concentrations.

These equations only apply when the concentration of one of the analytes substantially exceeds the other, as in the Bossegraben. If both [iP] and [As(V)] are expected to be high, using As(V) standards in high acidity is recommended, due to possible interference (Supp. Figure S2), changing eqs 1 and 2 to
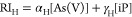
4
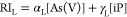
5

Scanned images of sample gels were superimposed with Image J and Adobe Illustrator in 8-bit format and then converted to data tables using imager 0.42.1^32^ for R 4.0.3.^33^ Values were first calibrated for overall color saturation, then for linear regressions per analyte, and imaged with ggplot2 3.3.5.^34^

### 2.4 Application in Soil

#### 2.4.1 Site and Sampling

The sampling site Bossegraben (51.901111°N, 10.498083°E) is a small brook near Oker, in the Harz, Germany. The brook flows along a mining deposit and was impacted by arsenic input before the beginning of remediation actions in the 1990s. Present-day arsenic concentrations in the water column may still reach up to ∼5 μM. Porewater arsenic concentrations were previously found to increase with depth in the reduced zone’s soil at the site sampled here (site 3 in Castillejos Sepúlveda et al.^15^). The soil is muddy, interspersed with small rocks, and rich in organic material. Geologic assays of the area surrounding the Bossegraben indicate the prevalence of sandstone and permeable limestone deposits (Fachbereich Bauen & Umwelt—Bodenschutz/Deponiemanagement and Fachbereich Umwelt und Gewässerschutz, Landkreis Goslar, personal communication, 2021).

Four soil cores (1 × 14 cm diameter by 14 cm height for gel probes, 3 × 5.4 cm diameter by 20 cm height for porewater extraction) were taken from the Bossegraben in May 2021. Cores were cooled on ice until processing at the Max Planck Institute in Bremen. Upon arrival, they were placed at room temperature with light airflow directed at the surface.

#### 2.4.2 Analyte Mapping by DETs

Probe preparation was adapted from Metzger et al.^19^ (Supp. Figure S1). Briefly, an opaque plastic holder (14 × 12, 0.3 cm thickness) was constructed with a rectangular depression (10 × 8 cm, 0.2 cm depth). Four polyacrylamide gels, soaked overnight in 750 μM SO_4_^2–^ to match the environmental concentration,^15^ were cut to 10 cm by 8 cm and stacked into the holder (Figure 1B). A clean membrane (hydrophilic PVDF, 0.2 μm, Durapore) was taped over the gels. Silver sheets were taped to the back of the probe for sulfide detection (Supp. Methods 1.3). The assembled probe was submerged in 750 μM SO_4_^2–^ and purged with N_2_ for 6.5 h, and then inserted vertically into a large soil core (14 cm ID) for 12.5 h (Figure 1C).

The deployment duration was based on previously published methods for the equilibrium of phosphate and iron, which reached equilibrium within 5 h in 0.46 mm gels.^7^ Since thicker gels were used here, time to equilibrium was calculated according to the modified Einstein diffusion equation
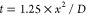
6where *x* is total gel thickness in mm, *D* is the diffusion coefficient in sediment, and *t* is time to reach equilibrium in seconds.^7^ Thus, for a stack of four gels (2 mm), the calculated time was 4.17 h. To ensure equilibrium was reached, at the expense of temporal sensitivity, equilibrium time was extended to 12.5 h.

Immediately after removal from the soil core, gels in the probe were separated and each gel layer was placed on a gel previously soaked with one of the following reagents: H, L, LO, or ferrozine and reducing reagent (Figure 1D,E). Before hyperspectral imaging, all gels were covered with a transparent sheet to hinder evaporation. Gels for iron analysis were scanned immediately (see Section 2.2), all other gels were scanned 2 h after contact time. Calibrations of As(V), As(III), and iP were performed 2 days before sample probe deployment, iron calibrations were performed the day of the sample probe deployment.

#### 2.4.3 Porewater Analysis

One day after the gel analysis, porewater was extracted every 0.5 cm with rhizones (Rhizosphere Research Products, NL, 0.25 cm diameter, 5 cm filter length) from the three small cores (5.4 cm ID). Immediately after extraction, total iron was analyzed according to Viollier et al.,^23^ and arsenic and iP were measured in triplicate following the SDS protocol from Castillejos Sepúlveda et al.^15^

#### 2.4.4 μXRF Imaging

A rectangular vertical soil sample (13 cm width by 10 cm height) was taken next to where gels were deployed and immediately frozen at −80 °C, and then freeze-dried. Resin (EPO-TEK MED-301-2FL) was applied under a vacuum from the bottom to the top. The resin-embedded soil was polished using 3 μm diamond paste.

An M4 Tornado μXRF spectrometer (Bruker Nano Analytics, Germany) equipped with a Rhodium X-ray source and polycapillary optics (20 μm spot size) was operated at 50 kV and 600 μA under vacuum condition of 20 mb for elemental mapping of the polished core slice. Pixel size was set to 100 μm and scan time to 5 and 30 ms/pix for total area overview and transects analyses with higher intensity, respectively (Supp. Methods 1.4). Elemental distributions were analyzed as net intensities (deconvoluted counts) using the M4 Tornado software.

## 3 Results and Discussion

### 3.1 Method Validation

**Figure 2:**
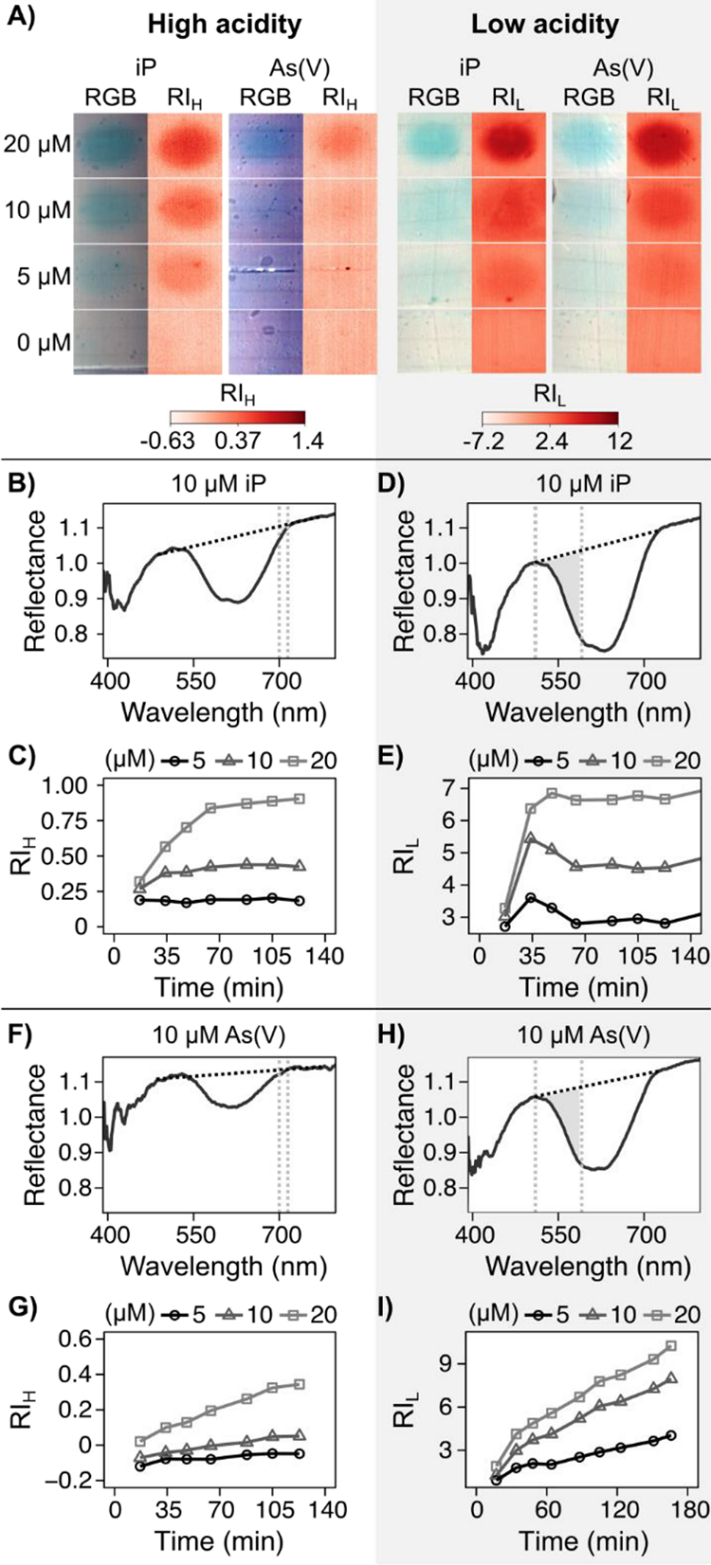
As(V) and iP detection in DET gels using the high (gel H) and low (gel L) acidity reagents. (A) images of the natural view and the index of reflectance (RI) of standards after two hours of reaction time. (B, D, F, H) Reflectance spectra from 400 to 780 nm of 10 μM As(V) and iP standards. Defining wavelengths are marked with vertical lines, the baseline is shown by a diagonal line, and the filled gray area represents the AUC used as RI. (B, C, F, G) In gel H, RI_H_ was calculated as the AUC from 700 to 715 nm with a baseline from 485 to 776 nm. (D, E, H, I) In gel L, RI_L_ was calculated as the AUC from 510 to 591 nm with a baseline from 510 to 725 nm. Full images of the calibrations, signal development over time, and regression models for As(V), As(III), and iP in all reagents can be found in Supp. Figures S2–S9. (C, E, G, I) Development of the measurement index over time in 5, 10, and 20 μM in As(V) and iP standards.

Detection of As(V), iP, and As(III) in calibration gels was consistent with the equations expected from previous cuvette measurements,^15^ outlined in eqs 1–3. Namely, iP could be detected in gels L and H (Figure 2A,B–E), while As(V) was almost negligible under 10 μM in gel H (Figure 2A,F,G). As(III) could only be measured after oxidation to As(V), thus only in gel LO (Supp. Figures S6 and S9). Consistent with the observations in Cesbron et al.,^16^ calibration gels and in situ gels showed well-defined 2D structures. Additionally, the RI did not decrease again after reaching its maximum (Figure 2C,E) suggesting minimal color loss due to diffusional relaxation. This results from the formation of stable molybdenum-iP and As(V) complexes. Due to their large size, these complexes have low coefficients of diffusion. Therefore, the loss of spatial information is minimized. Less spatial accuracy is expected for As(III), since it can diffuse laterally through the gel until oxidation is complete.

Measurement accuracy for all analytes was strongly dependent on the combination of RI used. RI were selected in each reagent gel to quantify the color development and to minimize the interference from high As(V) in gel H (Figure 2). Initially, RIs were calculated based on RGB wavelengths (Supp. Results 2.1). Concentration mapping based on ratios of RGB wavelengths was accurate for As(V) and iP but not for As(III) (Table 1 and Supp. Figure S10). However, accurate results could be obtained through hyperspectral imaging. The most accurate RI were: (RI_H_) AUC between 700 nm and 715 nm, with a baseline from 485 to 776 nm (Figures 2B,C,F and S2B, S3B), (RI_L_) AUC between 510 and 591 nm, with a baseline from 510 to 725 nm (Figures 2D,H and S4B, S5B, S6B), (RI_LO_) AUC between 600 and 675 nm, with a baseline from 596 to 710 nm (Supp. Figures S7B, S8B, and S9B). Using these measurements, calibrations performed in both ultrapure water and ASW yielded linear regression equations up to 25 μM for As(V), and 20 μM for iP and As(III) (Supp. Figures S2–S9, ASW not shown). Values below 2.5 μM were indistinguishable from zero (Supp. Figure S11).

**Table 1:** Total and Percent Recovery of As(V), As(III), and in iP Mixed Samples Prepared in Ultrapure Water^a^

	[As(V)] = 6.7 μM, [iP] = 6.7 μM, [As(III)] = 6.7 μM		[As(V)] = 6.7 μM, [iP] = 13.3 μM		[As(V)] = 13.3 μM, [iP] = 6.7 μM	
	multiple spectra	RGB ratio	multiple spectra	RGB ratio	multiple spectra	RGB ratio
As(V)	7.4 μM (110%)	7.6 μM (114%)	5.8 μM (88%)	6.3 μM (95%)	10.1 μM (76%)	10.2 μM (77%)
iP	6.7 μM (101%)	7.2 μM (109%)	12.2 μM (92%)	12.6 μM (95%)	7.3 μM (110%)	6.3 μM (94%)
As(III)	5.8 μM (87%)	–3.6 μM (−54%)				

a Calculations were based on the following: for both sets of equations, the gel H was measured at 1 h of reaction time, the gel L at 2:45 h, and gel LO at 2:45 h of reaction time. RGB ratio: *R*_640_ divided by *R*_460_ for all reagents; multiple spectra: (RI_H_) area under the curve between 700 and 715 nm, with a baseline from 485 to 776 nm, (RI_L_) area under the curve between 510 and 591 nm, with a baseline from 510 to 725 nm, (RI_LO_) area under the curve between 600 and 675 nm, with a baseline from 596 to 710 nm.

While the reaction was incomplete after 3 hours, dependency of RI on concentration was linear throughout (Supp. Figures S2–S9). Accuracy was increased when gel H was scanned after 1 hour of reaction time, and gels L and LO were scanned after 2.75 h (Table 1). Standards and sample gels used for the Harz soil were all scanned two hours to minimize the loss of information of 2D arsenic-containing structures in gels L and LO (Supp. Methods 1.1). Given the low and extremely localized distribution of iP, extending the reaction time for gel H did not lead to major disruptions of spatial information. However, in samples with more spatial overlap of arsenic and phosphate, minimizing reaction time of all gels would be essential to preserving 2D information.

Recovery of mixtures of As(V), As(III), and iP in ultrapure water and ASW is summarized in Tables 1 and S1. Recovery of concentrations in mixed ultrapure water standards was in the range of 77–114% for As(V) and As(III), and 94–109% for iP. In ASW, recovery ranges were less satisfactory. Therefore, further optimization is needed for application in saline water.

To summarize, this method is suitable for freshwater systems with less than 25 μM of each of iP, As(III), and As(V). Previous uses of this colorimetric method showed no interference from iron (up to 100 μM), sulfide (up to 100 μM), or organic matter as in the Bossegraben samples.^15^ Other suitable applications include root systems of plants, submerged microbial mats, or contaminated lake sediment. Sites need to be shielded from vigorous mixing, such as strong currents or animal movement, to allow the gels to reach equilibrium. This can be avoided by sampling larger cores of soil or sediment, as done here.

### 3.2 Comparison of Analyte Determination by DETs to Traditional Porewater Extraction in the Bossegraben

**Figure 3:**
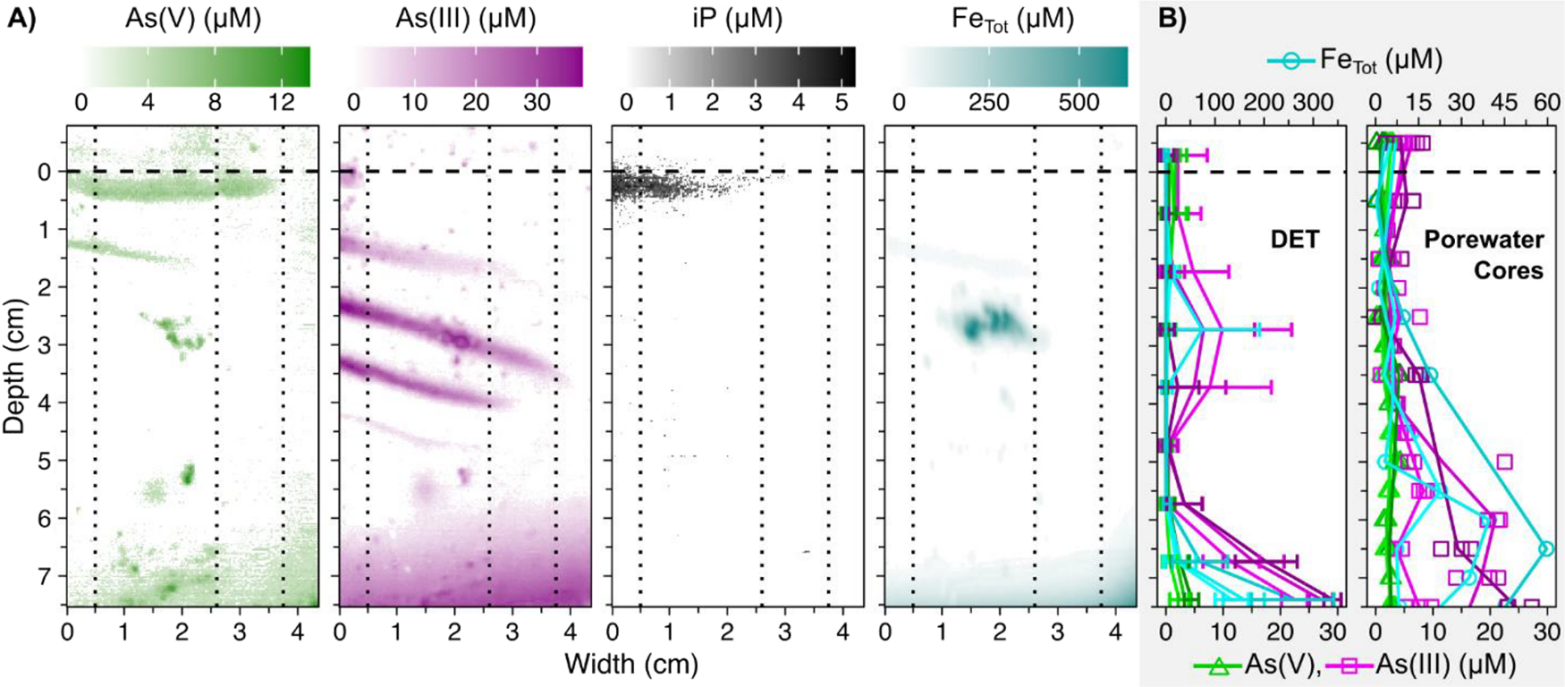
Concentration profiles over width of As(V), As(III), iP, and total iron in natural soil from the Bossegraben brook in the Harz mountains, Germany, in gels (A) and depth profiles (B). The approximate soil surface is indicated by a black dotted line. (A) For As(V), As(III), and iP, values were determined based on multivariate models derived from the linear regressions of the three analytes across reagents with differing acidities. Values above 25 μM in As(III) represented an oversaturation of analyte on the reagent gel and may not be accurately determined. Concentration depth profiles of measured As(V) (green), As(III) (magenta), iP (black), and total iron (blue). (B) Arsenic and iron profiles labeled “DET” were calculated from color-reacted gels (vertical dashed lines in gel images), simulating porewater extraction. Values shown are averaged over sections of 1 cm depth and 1 cm from the central width indicated for each profile (0.5, 2.6, and 3.75 cm). Error bars were calculated using standard deviation. Profiles labeled ‘Porewater Cores’ show concentrations in porewater extracted from three soil cores. For porewater extraction, technical replicates = 3 per core for As(V), As(III), iP; 1 per core for total iron.

DET imaging revealed the distribution of all analytes in a distinct banding pattern, likely linked to layering of leaves and/or variable deposition, as well as a prominent patch of arsenic and iron at 2.5 cm depth. Traditional porewater measurements failed to reflect the mm-scale banding pattern due to the limited resolution of porewater extraction (Figure 3).

To confirm that the differences between DET and extracted porewater results were not an artifact, we predicted the results of porewater extraction based on the DET images. Specifically, we calculated average concentrations over depth (1 cm due to smearing) and width (1 cm). The resulting average depth profiles of As(V) and As(III) were in great agreement with the traditional method (Figure 3B). However, iron concentration in extracted porewater increased with depth in two out of three cores, indicating reductive dissolution of iron oxides as previously suggested,^15^ which was not well represented in the gels (Figure 3A). This could have several reasons, such as iron oxidation. Especially in porewater extraction, iron oxidation would additionally lead to an underestimation of [As(V)] and [iP] due to their sorption onto iron oxides. This was not the case using gels. The gels remain anoxic during sampling, and are only oxidized when retrieved. Therefore, analytes are already contained in the gel matrix and do not change in position or concentration between sampling and contact with the reagent gel. An alternative explanation for the differences in Fe depth distribution is that sensitivity may have been insufficient. This could be targeted using thinner reagent gels with higher reagent concentration to minimize sample dilution. Since not all cores clearly showed increasing Fe with depth, lateral heterogeneity is the most likely explanation for the differences between Fe in DETs and in extracted porewater.

### 3.3 Localized Release of Arsenic into the Bossegraben Water Column

As(III) occurred in four distinct bands reaching down to 5 cm. Except for the central patch, As(V) found in patches below 0.5 cm was surrounded by As(III), as seen at 1.25 cm depth, and small patches at the bottom of the gel (Figure 3A). While As(III) was therefore not in exchange with the water column, an As(V)- and iP-rich band was localized directly under the surface (Figure 3 and Supp. Results 2.2). The corresponding steep gradients across the soil–water interface suggest release and net export of As(V) from the soil (Supp. Results 2.3). Previous measurements of water column arsenic showed a decrease in porewater and water column arsenic concentration downstream from the study site. It is thus unlikely that local leaching has a broader environmental impact,^15^ although downstream dilution by an external water source remains a possibility.

### 3.4 Displacement of iP by As(V)

Due to the similarity between As(V) and iP, As(V) can compete with iP for binding sites^35^ such as the surface of metal oxides, which form upon contact with oxygen at the surface of the soil. Thus, it is possible that iP in the Bossegraben was released due to displacement by As(V) or vice versa.^36^ iP was only measured in gels, in a single band close to the surface which also coincided with a larger As(V) band (Supp. Figure S12). No iron was measured in this band. As(III) was only present in Section 2, and was not strongly correlated to iP (*p* = 0.4, *R*^2^ = 0, Supp. Figure 12B). However, analysis of As(V) and iP in this band show distinct patterns of negative correlation between them (*p* < 0.001, *R*^2^ > 0.75), in agreement with displacement of sorbed species. Given that As(V) was also found above the surface of the soil, it is more likely that As(V) was present as a solute, displacing iP. Upon sorption onto the minerals at the surface, As(V) could have slowly replaced iP, leading to the localized release of iP.

Preferential bonding of As(V), rather than iP, to minerals was also supported by correlation analyses over the entire depth of the gel. Although iP was scarce under 1 cm depth, most of the iP measured did not overlap with the presence of total iron (Supp. Figure S13). Correlations between both iP and total iron were not statistically significant in any of the areas analyzed (*p* > 0.5 in all, except in boxes 5 and 7 where *p* < 0.5 but *R*^2^ = 0, Supp. Table S2). Thus, it is unlikely that iP was bound to iron particles.

The low iP concentrations at depth could also be related to a general lack of iP release from buried minerals. This would be in agreement with the burial origin of iron- and arsenic-containing minerals. Namely, these particles would have slowly been buried over a long period of time before remediation measures in the site were undertaken. During this slow process, As(V) displacement of iP would have taken place simultaneously with burial. Thus, the surface of present-day minerals would be depleted of iP, unless more iP was provided or displacement by As(V) was hindered. Our results showed that arsenic was mostly found in its reduced form under the surface, although processes like biotic As(III) oxidation could be a source of As(V) at depth. Therefore As(V) displacement would be minimized at depth in the present day. Input of iP is expected from bacterial breakdown of organic matter, such as from the abundant vegetation surrounding the Bossegraben. Although high iP concentrations would thus be expected in this site, the very low concentrations measured may indicate it is quickly taken up by plants or bound to dissolved organic compounds (i.e., humic acids). The former may be of concern considering the similarity of As(V) and iP and unspecificity of phosphate transporters, like those from the Pht1 phosphate transporter family, in some plants.^37−39^ Overall, the suspicions limitation of iP abundance to the uppermost layers substantially differs from established scenarios in soils and sediments, in which iron oxides in surface layers trap this nutrient.^40^ This is likely facilitated by the overwhelming abundance of As(V) competing for sorption sites.

### 3.5 Abundant Mineral-Sorbed Arsenic in Soil

**Figure 4:**
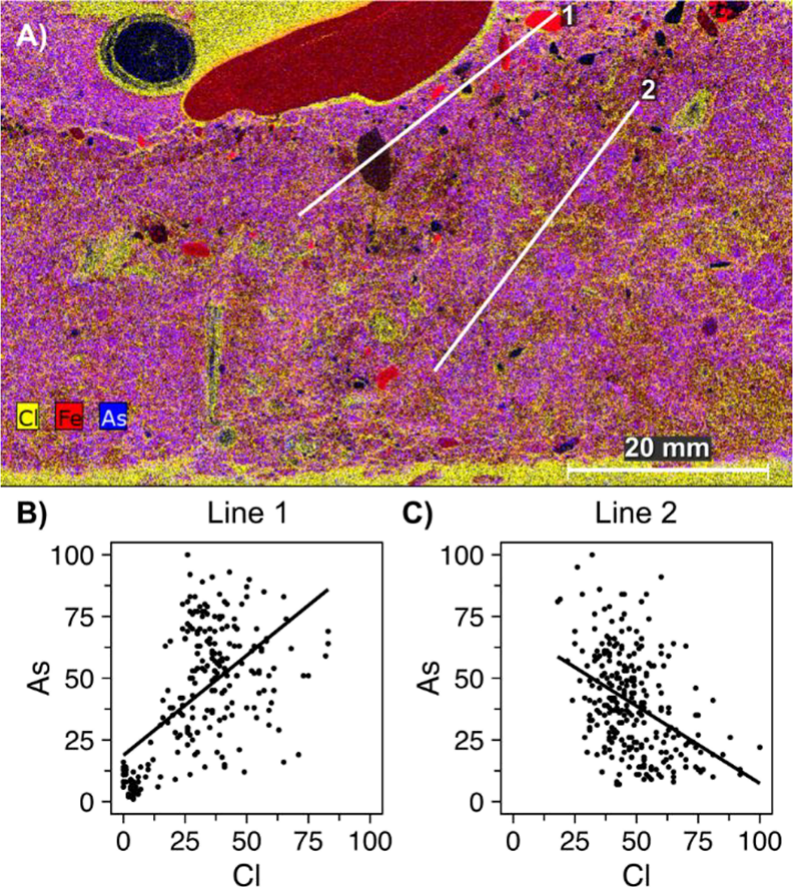
(A) Superimposed μXRF image of a slice of Bossegraben soil, showing chlorine (yellow), iron (red), and arsenic (blue) in the solid phase. Lines 1 and 2 indicate subsections used for analyses. All analyte peaks were deconvoluted. (B and C) Correlation of relative counts of arsenic and chlorine in line 1 (*R*^2^ = 0.35, *p* < 0.001) and line 2 (*R*^2^ = 0.018, *p* < 0.001). Chlorine can be used as an indicator of porewater space as it is abundant in the resin used for fixation. A positive correlation of As with Cl thus indicates increasing arsenic presence with increasing porewater, while a negative correlation indicates decreasing arsenic presence in the porewater. Further analyses on iron and arsenic distribution may be found in Supp. Results 2.4–2.5, scans of individual elements may be found in Supp. Figures S15–S18.

Arsenic release into the porewater, and ultimately the water column, is likely driven by dissolution of solid-phase arsenic. The patch at 2.5 cm depth (Figure 3) likely resulted from a solid particle rich in arsenic and iron, pressed directly against the gel, as evidenced by its sharp outline. Additionally, the μXRF analyses showed abundant patterns of arsenic and iron throughout the soil (Figures 4 and S14–S19).

The depth-dependent distribution of arsenic species may be explicable based on the history of arsenic transfer into the system. Arsenic in the soil could derive from two sources: (1) historical and/or ongoing input of dissolved arsenic carried by runoff from the deposit, which could then enter water column of the Bossegraben, and (2) particle transport from the waste deposit area in the period of time before remediation measures were undertaken.^15^ Due to the high abundance of particle-associated arsenic and iron at depth, both in the central patch of DET gels and in line 2 of μXRF images (Figure 4), arsenic at depth likely originated from source 2 before undergoing burial. Solid-phase arsenic within iron layers could still partially be bound to minerals, due to low direct exposure to reducing conditions leading to mineral dissolution.^2^ Additionally, dissolved arsenic from source 1 likely adsorbed onto the surface of the iron-rich minerals^27,41^ before remediation. Close to the surface, arsenic is increasingly present in the pore space rather than in solid particles (line 1, Figures 4 and S14), which could indicate relatively new input from an As(V)-rich water column, or result from upward transport (likely diffusive) of arsenic after release in the deeper layers.

### 3.6 Local Desorption Processes and Redox Transformations

**Figure 5:**
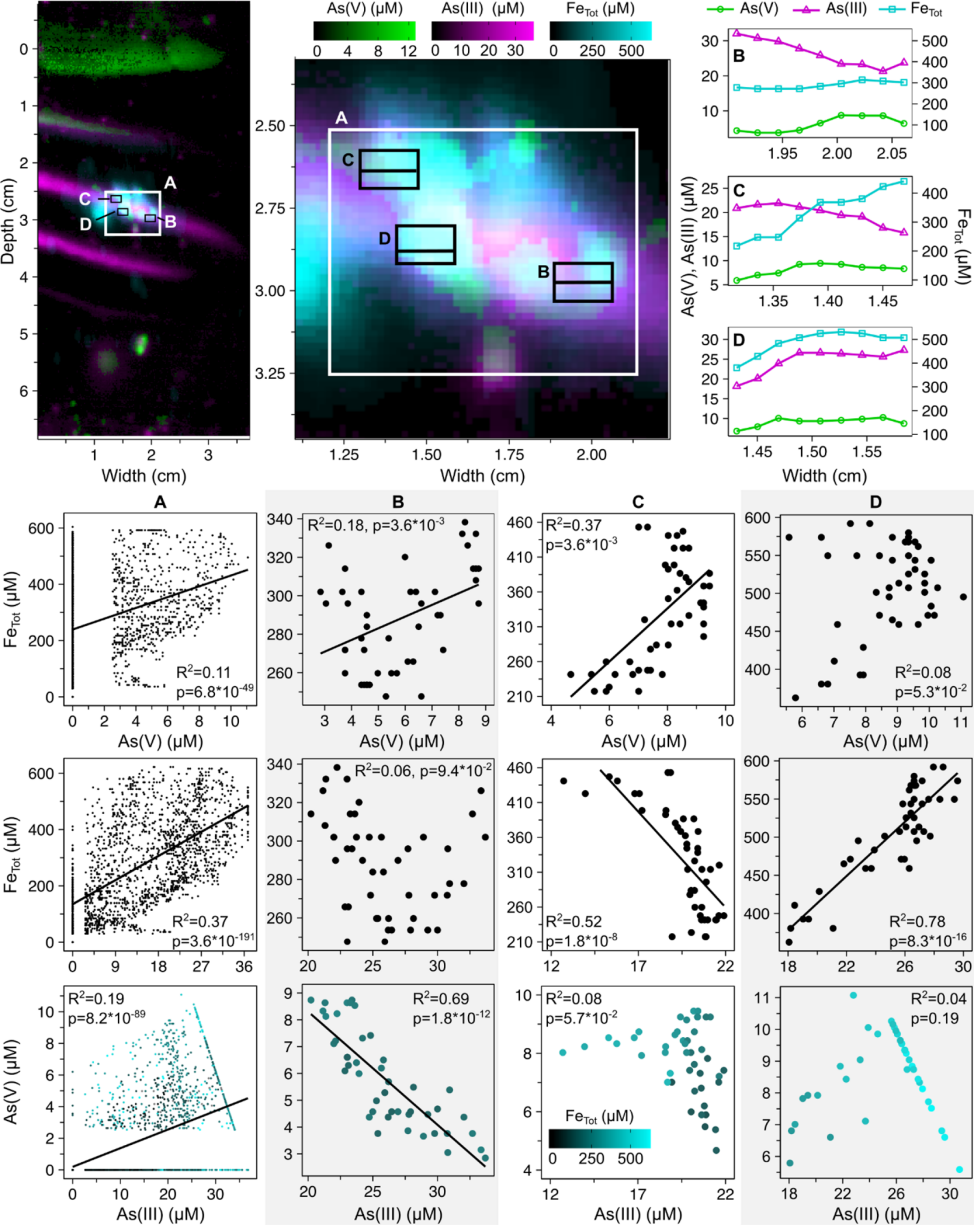
Correlation of As(V) (green) and As(III) (magenta) species to total iron (blue) detected in gel images and between As(V) and As(III) on a subsection of As deposition (A). Horizontal bars denote the location of the concentration-width profiles for B, C, and D. As(III) values above 25 μM may not be accurate. Correlation plots were calculated using all pixels within the boxes for each of A, B, C, and D. Linear regressions are shown for *p* > 0.01. All regression equations, *p*, and *R*^2^ values can be found in Supp. Table S2.

Iron concentration in the extracted porewater was low and mostly below detection limit in gels, except for a central patch (see box A in Figure 5) likely representing porewater around a particle undergoing reductive dissolution. Pixel-by-pixel correlation at different locations within the patch showed apparent co-occurrence of iron, As(III), and As(V) (Figure 5). Over a relatively large area (Figure 5A), As(V) and As(III) were mostly present in high concentrations when iron was also in high concentrations (>100 μM) (see correlation plots in Figure 5A). Inspection of three smaller locations showed pronounced heterogeneity of As(V), As(III) and iron distribution patterns. Subsections were selected based on proximity to well-defined microstructures, while avoiding most of the color signal oversaturation (Figure 5).

In the first location (Figure 5B), there was a significant (*p* = 3.6 × 10^–3^) correlation between As(V) and iron, but not between As(III) and iron. The second location contained an iron hotspot (>410 μM). Further away from this particle, iron was also positively correlated to As(V), (*p* = 1.0 × 10^–5^), but negatively to As(III) (*p* = 1.8 × 10^–8^). The decrease of As(V) may indicate eventual reduction to As(III), which accumulates with distance to the hotspot. In contrast, As(III) and iron were strongly positively correlated (*R*^2^ = 0.78, 8.3 × 10^–16^) in the third location. The correlation between As(III) and As(V) also appeared to be positive, until data was obscured by the upper concentration limit of the colorimetric method for arsenic measurement. These fine-scale correlation analyses clearly highlight the advantage of using the combination of DET with colorimetry, as most other assays would have obscured these microenvironmental processes.

The emergent picture based on the DET and μXRF data is that of arsenic leaching driven by reductive dissolution of iron oxides, and release of sorbed As(V) in deeper soil layers (Figure 5). Released As(V) is oxidized to As(III) by microbially mediated or abiotic processes at a spatial and thus temporal offset.^42^ These processes are evidenced by the negative correlations between As(V) and As(III) in two of the locations in the gels, the lack of evidence for dissolved As(V) in the porewater band, and the identification of arsenic associated with solid-phase iron in the μXRF images (Supp. Figure S14C,E). The origin of Fe-sorbed As(V) remains obscure. Most likely, iron-sorbed As(V) particles were simply transported to the soil before remediation and then buried. Alternative scenarios of historical redox transformations are also possible. Namely, iron-associated As(V) may have originated from dissolved As(III) supplied by runoff, before the sealing of the deposit in the 1990s. Porewater As(III) may have sorbed to iron oxides.^27,43^ Over time, microbial activity, e.g., denitrification coupled to As(III) oxidation,^44^ could have oxidized iron-bound As(III) (Figure 5B). In line with active arsenic redox cycling close to dissolving iron minerals, the positive correlation between As(III) and As(V) in section three would represent a snapshot of a more mature and reduced state.

**Figure 6:**
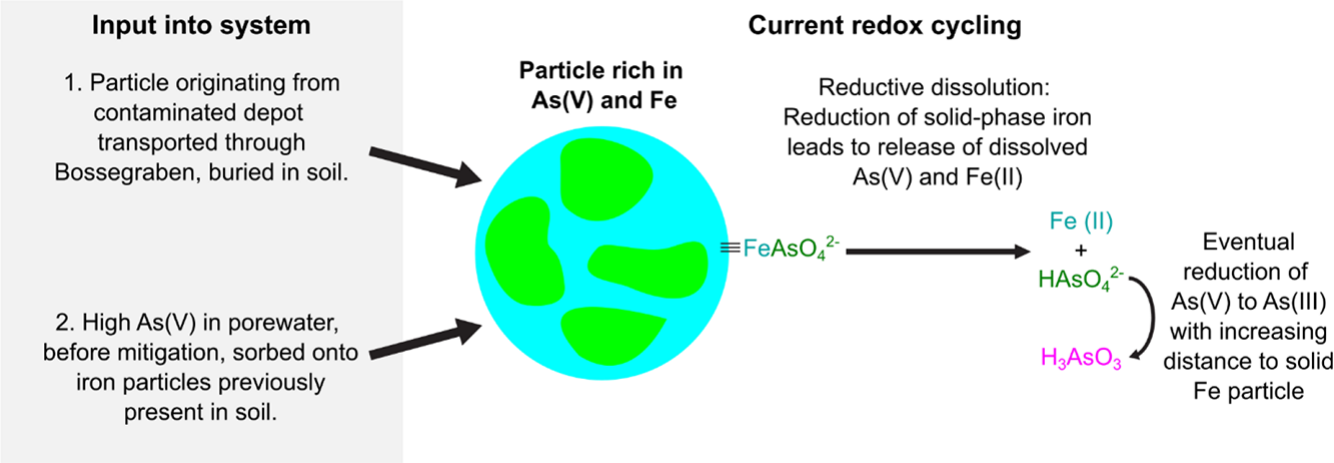
Proposed mechanisms involved in the cycling of iron and arsenic in minerals of the Bossegraben soil. As(V)-containing species are colored in green, As(III) in magenta, and Fe in blue.

In summary, DET imaging allowed for a quantitative estimation of arsenic leaching from the Bossegraben soil, for identifying a potential historical source, and for proposing spatially-resolved mechanisms of ongoing arsenic release and redox cycling. Specifically, current arsenic cycling likely occurs as result of reductive iron dissolution releasing iron and As(V) into the porewater (Figure 6). Released As(V) is subsequently reduced, then As(III) moves upward (through capillary action or diffusion), and is re-oxidized to As(V) before reaching the water column. Binding of As(V) to oxidized minerals at the surface could then lead to the localized release of iP.

While the prevalence of reduced As(III) over depth was expected, DET imaging allowed the capture of microscale trends that would have been otherwise overlooked. Future combinations with simultaneous mapping of pH and analysis of the redox state of undisturbed iron-bound arsenic (e.g., by EXAFS, XANES), may further clarify the mechanisms shaping the patterns observed.

Complementary techniques like cyclic voltammetry may also be used to target speciation dynamics difficult to capture by colorimetry, including those of sulfur and organic compounds. While no sulfide was detected during deployment (using silver sheets for trapping, Supp. Figure S20, the potential role of organic substances, which are known to play a major role in environmental arsenic cycling, cannot be excluded. Dissolved organic acids could capture a portion of free dissolved arsenic, preventing binding to solid minerals, like iron.^45,46^ Thus, arsenic trapped in organic acids could be more easily transported. Lastly, organic matter availability would directly impact arsenic release, since bacteria require organic matter for reductive dissolution. Future studies on rates of arsenic cycling in incubations may resolve the role of organics in the Bossegraben.

### 3.7 Advantages of Arsenic Imaging by DET Gels

The 2D images obtained through our applied colorimetric method show the high-resolution distribution of the dominant dissolved inorganic arsenic species, As(V) and As(III). Results may be comparable to other methods which do not directly provide data in two dimensions, like traditional porewater analysis and voltammetric electrodes, with the additional advantage of examining both cm- and sub-mm scale trends. Our method exploits the spectral resolution of hyperspectral imaging to enable simultaneous mapping of dissolved iP, As(V), and As(III) at a sub-millimeter resolution (∼0.2 mm per pixel).

This method is a powerful tool to study arsenic cycling at high resolution, capable of deepening our understanding of local redox transformations in soil systems, with potential applications in complex environments for the examination of arsenic cycling in root systems.

## Supporting Information Available

Additional details of materials and methods, results, and discussion, including details from analyses using RGB wavelengths, further μXRF images, and the full correlation parameters from Figures 3 and 5 (PDF)
