# Supplementary material for: Two-Dimensional Mapping of Arsenic Concentration and Speciation with Diffusive Equilibrium in Thin-Film Gels
Source: Environ Sci Technol. 2023 May 16;57(21):8107–17. doi: 10.1021/acs.est.3c00887 (PMC10233794; doi:10.1021/acs.est.3c00887)
Supplement: Supplementary file 1 — es3c00887_si_001.pdf [file es3c00887_si_001.pdf]

Two-dimensional mapping of arsenic concentration and speciation with diffusive equilibrium in thin-film (DET) gels

Andrea Castillejos Sepúlveda[1], Edouard Metzger[2], Sten Littmann[3], Heidi Taubner[4], Arjun Chennu[5], Lais Gatti[1], Dirk de Beer[1], Judith M. Klatt[1,6,7,8*]*

[1]Max Planck Institute for Marine Microbiology, Microsensor Group, Celsiusstraße 1, 28359, Bremen, Germany

[2]Laboratoire de Planétologie et Géosciences, CNRS, Université d'Angers, Nantes Université, Le Mans Université, Angers, France

[3]Max Planck Institute for Marine Microbiology, Biogeochemistry Group, Celsiusstraße 1, 28359, Bremen, Germany

[4]MARUM Center for Marine Environmental Science and Faculty of Geosciences, Organic Geochemistry Group, University of Bremen, Leobener Str. 8, 28359, Bremen, Germany.

[5]Leibniz Centre for Tropical Marine Research, Data Science and Technology, Fahrenheitstr. 6, 28359 Bremen, Germany

[6]Microcosm Earth Center, Max Planck Institute for Terrestrial Microbiology and Philipps-Universität Marburg, Germany.

[7]Center for Synthetic Microbiology (SYNMIKRO), Marburg, Germany.

[8]Biogeochemistry Group, Department of Chemistry, Philipps-Universität Marburg, Germany

Supporting Information

Table of Contents

1. Supplement to Materials and Methods 1
1.1 Reaction time optimization 2
1.2 Gel composition 2
1.3 Sulfide detection with silver plates 3
1.4 µXRF imaging 3
2. Supplement to Results and Discussion 3
2.1.Gel calibrations using RGB wavelengths 3
2.2 Phosphate distribution 4
2.3 Arsenic fluxes at soil surface 4
2.4 µXRF imaging 4
2.5 Microscale variation in chemical composition 6
3. Supplement to Figures 6
4. Supplement to Tables 21
5. Supplement to References 25

Total Pages: S1-S25

Supplementary Figures: S1-S20

Supplementary Tables: S1-S2

1. Supplement to Materials and Methods

1.1 Reaction time optimization

Extended reaction time is inadequate for application in gels due to diffusional relaxation[1]. Diffusional relaxation (or *lateral diffusional relaxation*) refers to the loss of two-dimensional precision as analytes diffuse laterally through the gel. In the cuvette-based protocol, only two sub-samples are used: the first is reacted with high acidity reagent and the second with low acidity reagent. The low acidity sub-sample is oxidized after the first measurement, thus doubling reaction time but limiting sample volume needed. However, for the DET approach, we use three separate samples and reagent gels (high acidity, low acidity and low acidity plus oxidant) and initiate oxidation immediately for the third gel. Since diffusion is dependent on particle size and charge, the amount of diffusional relaxation is dependent on the formation of a colorimetric complex. The iron-ferrozine color complex is able to diffuse through the gel, and thus microstructures of reduced iron may appear bigger than they actually were[2]. The phosphomolybdate complex only very slowly diffuses laterally due to its size[2], which must also be true for arsenomolybdate complexes due to the structural similarity between iP and As(V). To avoid lateral diffusion of As(III), which does not form a complex with molybdenum and is thus free to diffuse before the oxidation reaction is initialized, we minimized reaction time by using three separate gels. Furthermore, we tested RI based on a large variety of a parameters, including single wavelengths, ratios, and integrals (area under the curve (AUC)).

1.2 Gel composition

Polyacrylamide gels were used for calibration and sample gels due to their higher resistance to abrasion. Agarose gels were used for the reagent gels, since hardening of the gels was observed after soaking polyacrylamide gels in the reagent mixture.

1.3 Sulfide detection with silver plates

Two thin sheets of silver (99.5% purity) were taped to the back of the assembled DET sampling probes in order to capture sulfide present in the Bossegraben. The plates were scanned before and after gel deployment, using identical settings on a Xerox flatbed scanner.

1.4 µXRF imaging

All µXRF scans were performed with the M4 Tornado system (Bruker Nano Analytics, Germany), operating at 50kV and 600 µA and vacuum condition of 20 mb. Transects were analyzed as line scans and objects within the enclosing mapped area. The area maps had the following settings of pixel size and scan time: Map 1) 100 µm and 5 ms/px for overview of total area of polished soil slice (Supp. Figs. S15-S18); Map 2) 100 µm and 30 ms/px for transect (line scan and object) analyses in enhanced intensity (Fig. 4, Supp. Fig. S14 & S19).

Elemental distributions were analyzed as net counts applying deconvolution with M4 Tornado Software. Map 2, enclosing the elements of further interest, was used for line scan and object analyses. Line scans were displayed as net counts in relative scaling of individual elements (data exported for further analysis). Profile line 1 has a length of 263 pixel; length of profile line 2 is 275 pixel; (Supp. Fig. S14B - E).

Selected areas of interest in the map (objects) were processed with standardless quantification (results of normalized wt.% of elements exported for further presentation). Three objects were analyzed: "Sel. 1" representing a solid domain with size of 312 pixel; "Sel. 2" representing a pore space domain with size of 564 pixel; "Sel. 3" representing a solid domain with size of 288 pixel; (Fig. S19 A, B).

2. Supplement to Results and Discussion

2.1. Gel calibrations using RGB wavelengths

Calibrations performed using all RGB wavelengths (640 nm, 550 nm, 460 nm), were unsatisfactory. The coefficients of determination (R^2) for all linear regressions obtained from standard concentration and RGB index were around 0.7. Therefore, a ratio was chosen between

the available wavelengths, calculated as reflectance at 640 nm (red) divided by reflectance at 460 nm (blue), hereafter called ‘RGB ratio’. The resulting linear regressions had significantly higher R^2 values, over 0.98 for As(V) and iP in gels L and H. However, As(III) cannot be determined based on RGB wavelengths, as the regression models of iP and As(III) standards in gel LO were not linear.

2.2 Phosphate distribution

iP was below detection limit in deeper soil layers, both in gels and porewater measurements, except for an upper band of iP, which coincided with a larger As(V) band. Thus, it is possible that iP is released due to displacement by As(V). Due to the similarity between As(V) and iP, As(V) can compete with iP for binding sites[3] like metal oxides, which form upon contact with oxygen at the surface of the soil. Given that As(V) was also found above the surface of the soil, it is likely that As(V) was present as a solute. Upon sorption onto the minerals at the surface, As(V) could have slowly displaced iP, leading to the localized release of iP. However, to form conclusions on phosphate-arsenic dynamics, further information is necessary.

2.3 Arsenic fluxes at soil surface

To assess the potential areal release rate of the soil, fluxes of As(III) and As(V) were calculated across the soil-water interface, using 195 depth profiles derived from the gel images (Fig. 3). The range of As(V) fluxes across the surface was -227.167 to 97.61 µmol m^{-2} day^{-1} (first quartile= -98.85, third quartile= -38.60, median= -67.15), -528.95 to 566.99 µmol m^{-2} day^{-1} (first quartile = -18.37, third quartile = 119.77, median= 53.87) for As(III), and -655.43 to 485.63 µmol m^{-2} day^{-1} (first quartile= -76.63, third quartile= 50.66 , median= -13.87) for total arsenic. The large spread of flux values arises from strong localization of arsenic at the surface (Fig. 3).

2.4 µXRF imaging

Closer inspection of two transects, line 1 and line 2 (Supp. Fig. S14 & S19), revealed that individual relative abundance of arsenic in the soil was dependent on depth and soil density.

The individual relative abundance of chlorine was used as a proxy for porewater, aiming at the differentiation between dissolved and particle-associated arsenic. This is because during preparation of the core for µXRF imaging, the soil was embedded in a resin. The embedding resin had high chlorine content, while the soil had low chlorine content. Since the embedding resin filled the porespaces in the soil, these could be later observed in the µXRF images as locations of high chlorine content. We assumed that elements originally present in the porewater remained in the same location after the soil was freeze-dried. These elements would have then been resuspended upon addition of the chlorine-rich resin. Therefore, a strong positive correlation to chlorine may indicate that an element was originally dissolved in porewater. Since the relative abundance of iron, and its correlation to arsenic, varied substantially due to the patchy distribution of iron, line 1 was separated into two sections (Supp. Fig. S14).

Section 1 of line 1 was characterized by a large silicon-rich particle, poor in iron, arsenic and chlorine. Surrounding the particle, while iron and arsenic were strongly correlated (R^2=0.82, Supp. Fig. S14C), the positive trend of arsenic with chlorine suggests the presence of arsenic with increasing porewater. In contrast, iron and arsenic were negatively correlated closer to the surface, in section 2. There, the negative correlation between chlorine and iron further indicated that iron was mostly present as solid particles, the largest of which could be clearly seen in the µXRF image (Fig. 4 & Supp. Fig. S14A). It can be concluded that close to the surface, although solid iron was present (potentially as iron oxides), arsenic was predominantly dissolved in the porewater and only to a much lesser extent associated to solids. However, in line 2 (Supp. Fig. S14E), deeper in the soil, the negative correlation between arsenic and chlorine indicated that arsenic was mostly associated to solids. Given the negative correlation to chlorine, and positive correlation to arsenic (R^2=0.64), iron was assumed to be predominantly associated with solids onto which arsenic could sorb.

2.5 Microscale variation in chemical composition

Exemplarily, the small-scale variation of major elements across a pore space (7 mm diagonal profile of three selections; Supp. Fig. S19A) is presented as relative element distributions of the three selections (Supp. Fig. S19B). The solid matrix region (selection 1 & 3 in Supp. Fig. S19B) is dominated by iron and silicon (ca 60%), while calcium and chlorine dominate (ca. 60%) the pore space domain in the center (selection 2 Supp. Fig. S19B). Additionally, arsenic (< 0.5%) and iron were lowest in the central porespace of the transect, indicating that either most arsenic and iron in the environment was present as solid, or that these particles were not resuspended during resin addition. Small spikes in relative counts surrounding porewater channels could originate from dissolved arsenic and iron that were deposited during the drying process, and are unlikely to be a result of microbial activity since neither arsenic nor iron appear to be 'preferred' at these locations.

3. Supplement to Figures

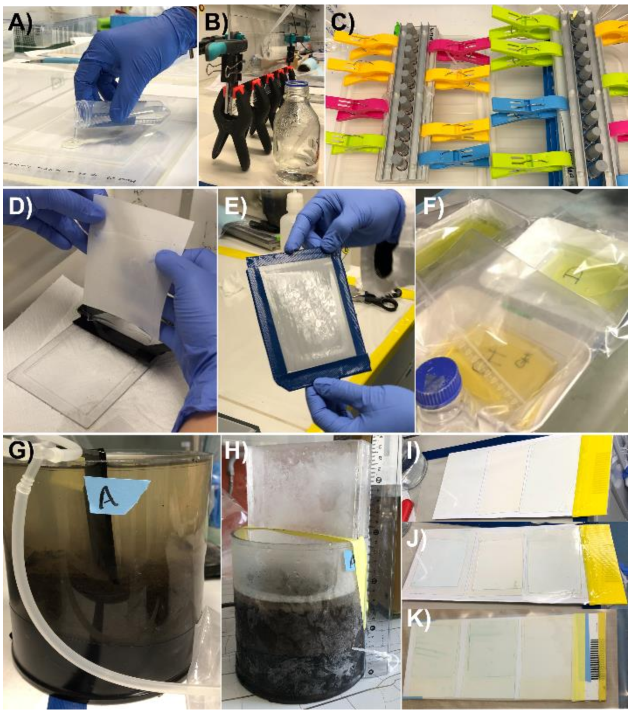

Figure S1. Photos of the experimental setup of DET gels, A) preparation of polyacrylamide 'sample' gels, B) preparation of agarose 'reagent' gels, C) setup of polyacrylamide calibration gels, D) preparation of sampling probes with polyacrylamide gels, E) prepared sampling probe, F) agarose gels soaking in reagent solutions, G) deployment of sampling probe in a core obtained from the Bossegraben brook in the Harz, H) rectangular core used to sample soil for

µXRF, frozen to facilitate removal, I) reagent gels placed on measurement board, J) sample gels immediately after placement on top of reagent gels, K) Bossegraben sample gels after 1 hour of reaction time (picture taken from separate samples than in I) and J)).

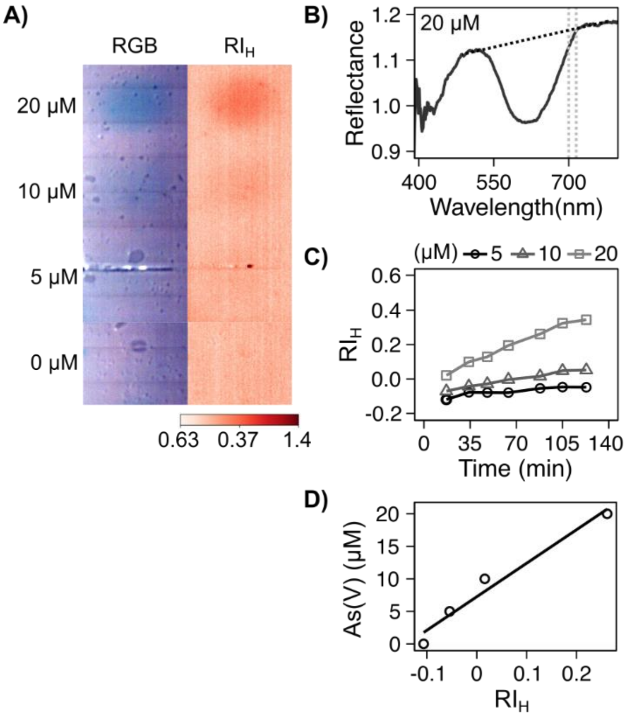

Figure S2. Calibration of As(V) detection using the high acidity reagent in DET gels. A) images of a regular scan view and hyperspectral view , with the index of reflectance (RI_H) calculated as the area under the curve (AUC) from 700 nm to 715 nm with a baseline from 485 nm to 776 nm) for each As(V) standard after two hours of reaction time. Scan view was edited using the settings lightness = 30.03 and contrast = 0.89, on Inkscape 1.1. B) Reflectance spectra of 20 µM As(V), filled gray area represents the measurement index, decisive wavelengths are marked with vertical lines, the baseline is shown by a diagonal line. C) Development of measurement signal over time for 5 µM, 10 µM and 20 µM As(V). D) Linear regression of the measurement index by standard concentration, after one hour of reaction time.

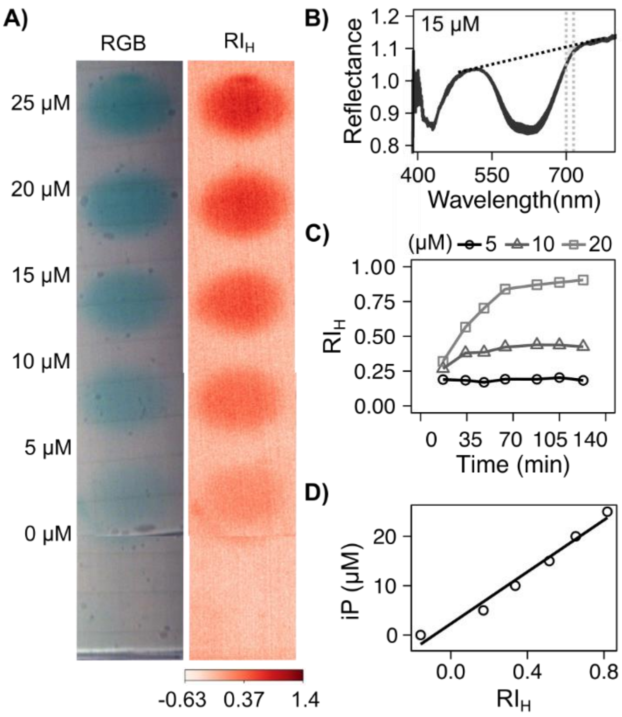

Figure S3. Calibration of iP detection using the high acidity reagent in DET gels. A) images of a regular scan view and hyperspectral view, with the index of reflectance (RI_H) calculated as the area under the curve (AUC) from 700 nm to 715 nm with a baseline from 485 nm to 776 nm, for each iP standard after two hours of reaction time. B) Reflectance spectra of 15 µM iP, filled gray area represents the measurement index, decisive wavelengths are marked with vertical lines, the baseline is shown by a diagonal line. C) Development of measurement index over time for 5 µM, 10 µM and 20 µM $_i$P. D) Linear regression of the measurement index by standard concentration, after one hour of reaction time.

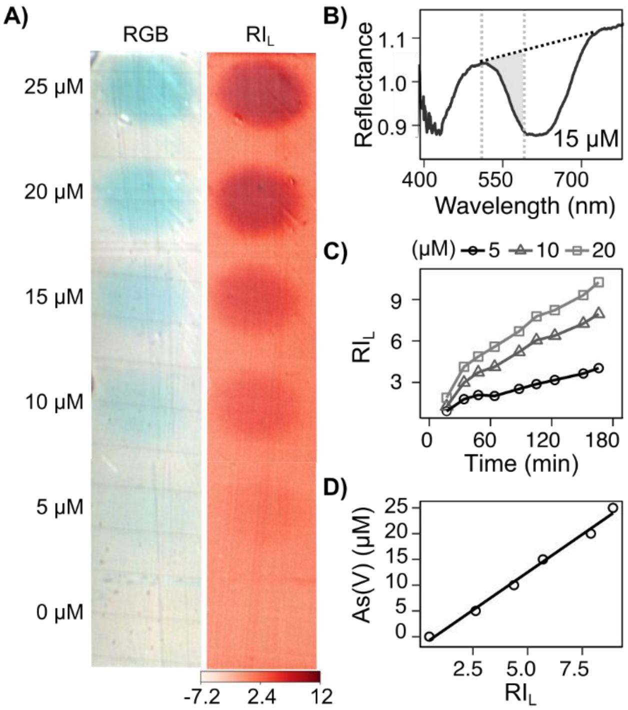

Figure S4. Calibration of As(V) detection using the low acidity reagent in DET gels. A) images of the natural view and the index of reflectance (RIL) calculated as the AUC from 510 nm to 591 nm with a baseline from 510 nm to 725 nm for each As(V) standard after two hours of reaction time. B) Reflectance spectra for 15 µM As(V) from 400 to 750 nm. Defining wavelengths are marked with vertical lines, baseline is shown by a diagonal line, and the filled gray area represents the measurement index AUC. C) Development of measurement index over time, in standards of 5 µM, 10 µM and 20 µM As(V). D) Linear regression of the measurement index against standard concentration, after 165 minutes of reaction time.

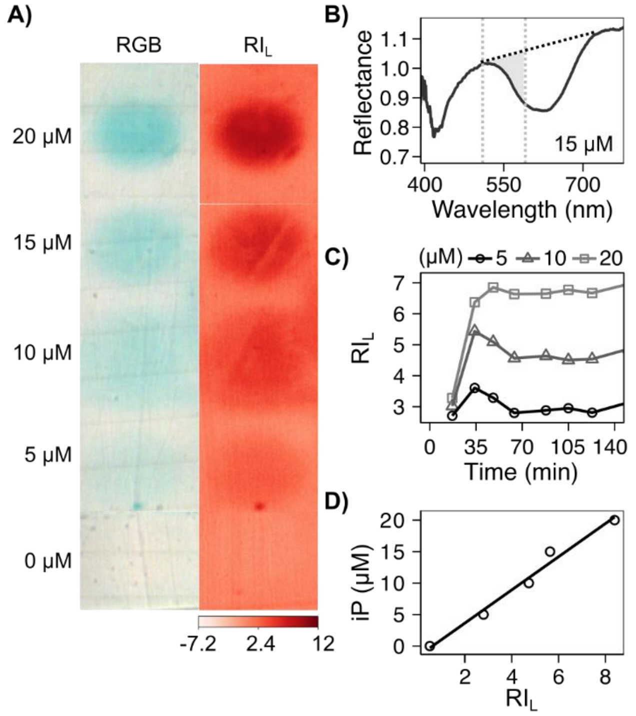

Figure S5. Calibration of iP detection using the low acidity reagent in DET gels. A) images of a regular scan view and hyperspectral view with the index of reflectance (RI_L) calculated as the area under the curve (AUC) from 510 nm to 591 nm with a baseline from 510 nm to 725 nm) for each iP standard after two hours of reaction time. B) Reflectance spectra of 15 µM iP, filled gray area represents the measurement index, decisive wavelengths are marked with vertical lines, the baseline is shown by a diagonal line. C) Development of measurement index over time for 5 µM, 10 µM and 20 µM iP. D) Linear regression of the measurement index by standard concentration, after 165 minutes of reaction time.

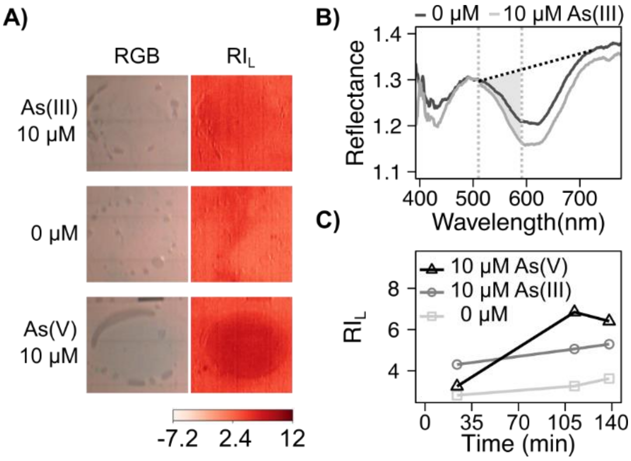

Figure S6. Calibration of As(III) detection using the low acidity reagent in DET gels. A) images of a regular scan view and hyperspectral view with the index of reflectance (RI_L) calculated as the area under the curve (AUC) from 600 nm to 675 nm with a baseline from 596 nm to 710 nm, for comparison to RI_{LO}, for a As(III) standard after two hours of reaction time. B) Reflectance spectra of 10 µM standard and a blank, filled gray area represents the measurement index, decisive wavelengths are marked with vertical lines, the baseline is shown by a diagonal line. C) Development of measurement index over time, after 165 minutes of reaction time.

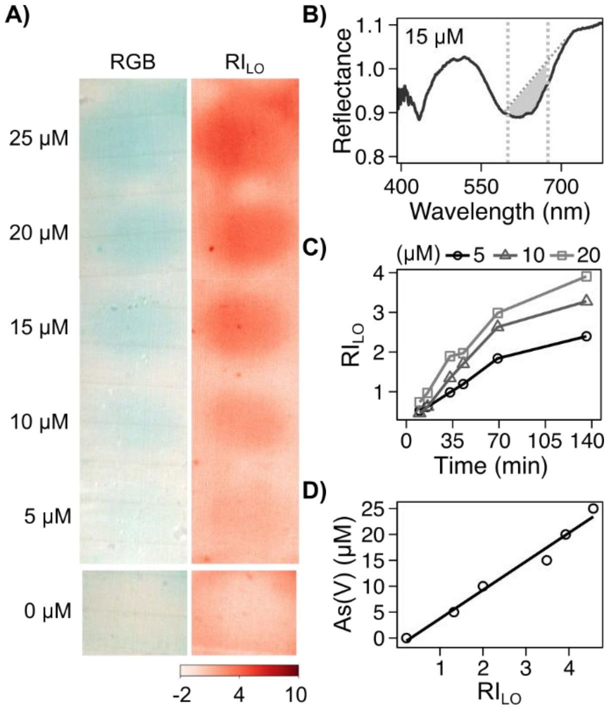

Figure S7. Calibration of As(V) detection using the low acidity + oxidant reagent in DET gels. A) images of a regular scan view and hyperspectral view with the index of reflectance (RI_{LO}) calculated as the area under the curve (AUC) from 600 nm to 675 nm with a baseline from 596 nm to 710 nm) for each As(V) standard after two hours of reaction time. B) Reflectance spectra of 15 µM As(V), filled gray area represents the measurement index, decisive wavelengths are marked with vertical lines, the baseline is shown by a diagonal line. C) Development of measurement signal over time for 5 µM, 10 µM and 20 µM As(V). D) Linear regression of the measurement index by standard concentration, after 140 minutes of reaction time.

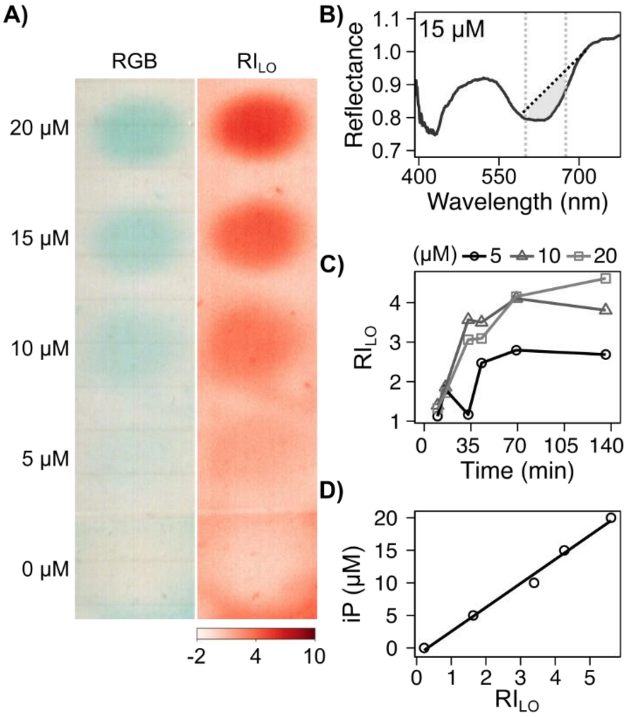

Figure S8. Calibration of iP detection using the low acidity + oxidant reagent in DET gels. A) images of a regular scan view and hyperspectral view with the index of reflectance (RI_{LO}) calculated as the area under the curve (AUC) from 600 nm to 675 nm with a baseline from 596 nm to 710 nm) for each iP standard after two hours of reaction time. B) Reflectance spectra of 15 µM iP, filled gray area represents the measurement index, decisive wavelengths are marked with vertical lines, the baseline is shown by a diagonal line. C) Development of measurement index over time for 5 µM, 10 µM and 20 µM iP. D) Linear regression of the measurement index by standard concentration, after 140 minutes of reaction time.

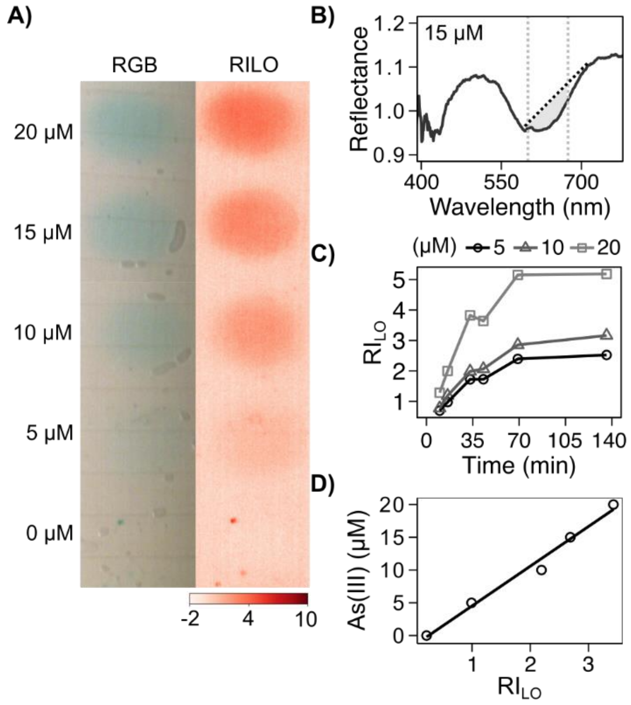

Figure S9. Calibration of As(III) detection using the low acidity + oxidant reagent in DET gels. A) images of a regular scan view and hyperspectral view with the index of reflectance (RI_{LO}) calculated as the area under the curve (AUC) from 600 nm to 675 nm with a baseline from 596 nm to 710 nm) for each As(III) standard after two hours of reaction time. B) Reflectance spectra of 15 µM As(III), filled gray area represents the measurement index, decisive wavelengths are marked with vertical lines, the baseline is shown by a diagonal line. C) Development of measurement signal over time for 5 µM, 10 µM and 20 µM As(III). D) Linear regression of the measurement index by standard concentration, after 140 minutes of reaction time.

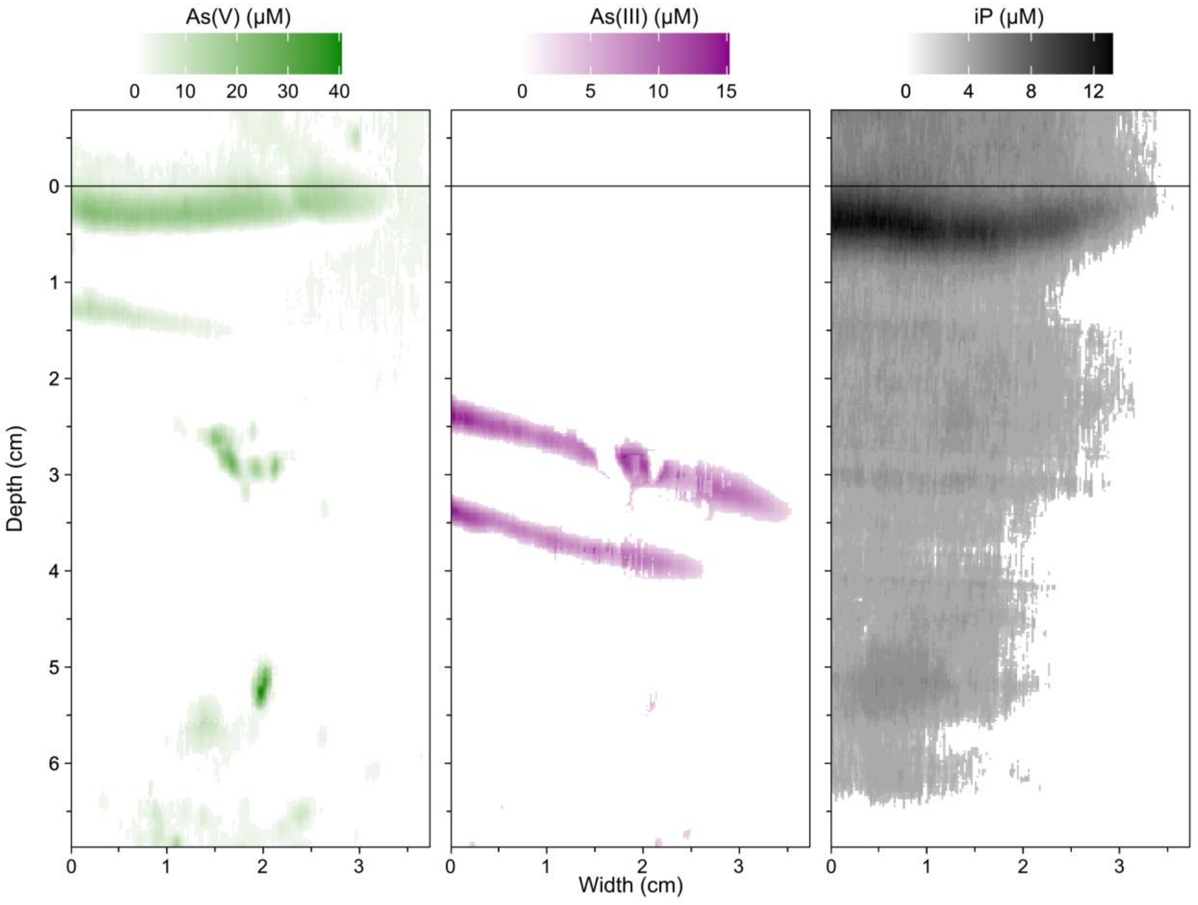

Figure S10. Predicted values of As(V), As(III) and iP in arsenic-contaminated soil in the Harz mountains. Approximate soil surface is indicated by a white dotted line. Values were calculated based on linear regression models of standard concentration to a ratio of $R_{640\ nm}$ to $R_{460\ nm}$, across three different reagents.

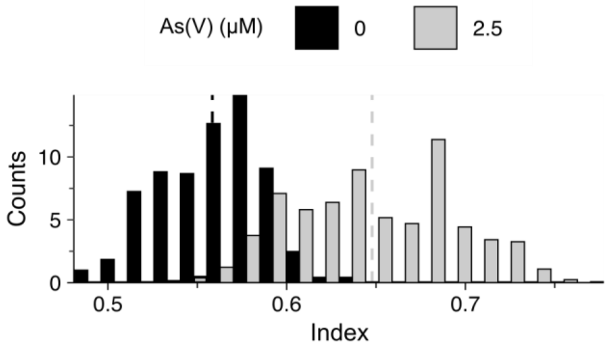

Figure S11. Counts of index per pixel in low acidity images of As(V) standards. The index of reflectance (RI_L) was calculated as the area under the curve (AUC) from 510 nm to 591 nm with a baseline from 510 nm to 725 nm.

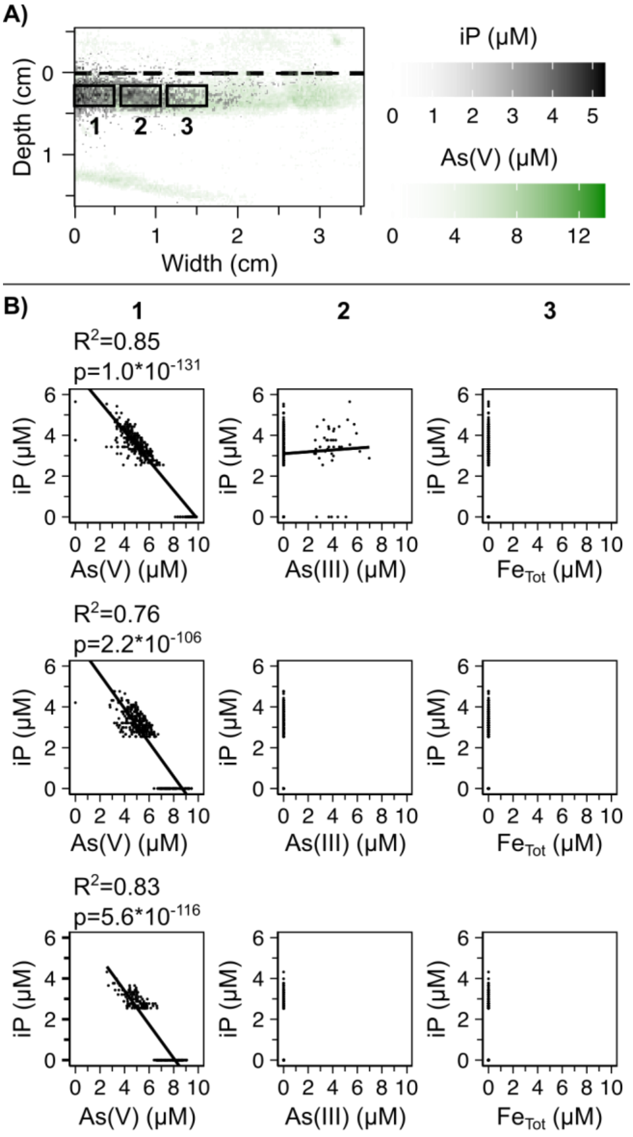

Figure S12. A) Correlation of As(V) (green) to inorganic phosphate (grayscale) detected in the upper band of the gel images. Values under 2.5 µM cannot be reliably distinguished from 0 using this method. Approximate soil surface is indicated by a black dotted line. B) Correlation plots between iP and As(V) calculated using all pixels within the boxes (1-3) in A. R^2 and P values shown for R^2>0.5. All regression equations, p, and R^2 values can be found in Supp. Table S2.

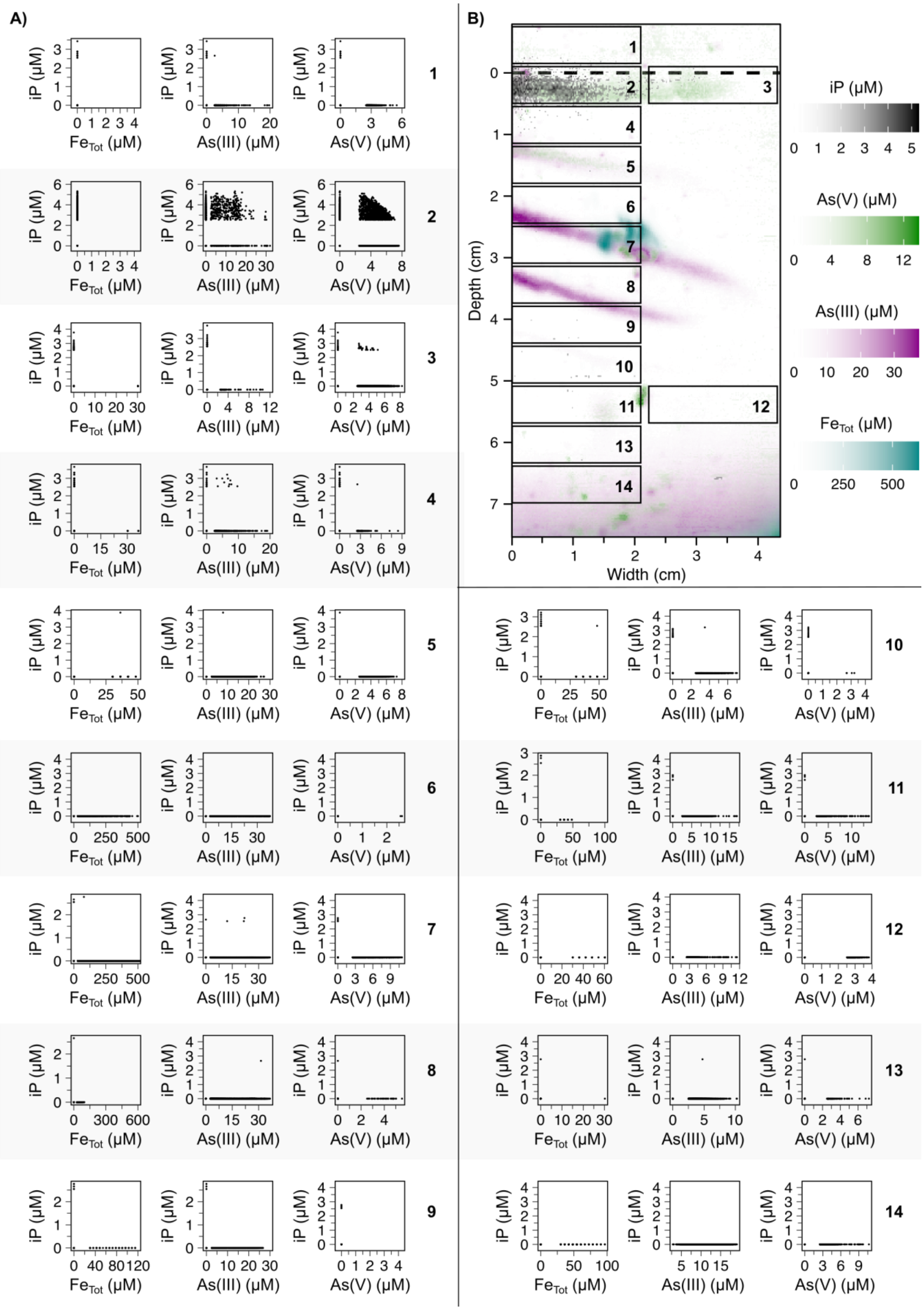

Figure S13. A) Correlations of inorganic phosphate to As(V), As(III), and total iron over large sections of the gel images. B) Areas chosen for analysis are depicted by black squares, parts of the gel without iP were not considered. Approximate soil surface is indicated by a black dotted

line. Values <2.5 µM could not be distinguished from zero, and were therefore considered as 0 µM. All regression equations, p, and R^2 values can be found in Supp. Table S2.

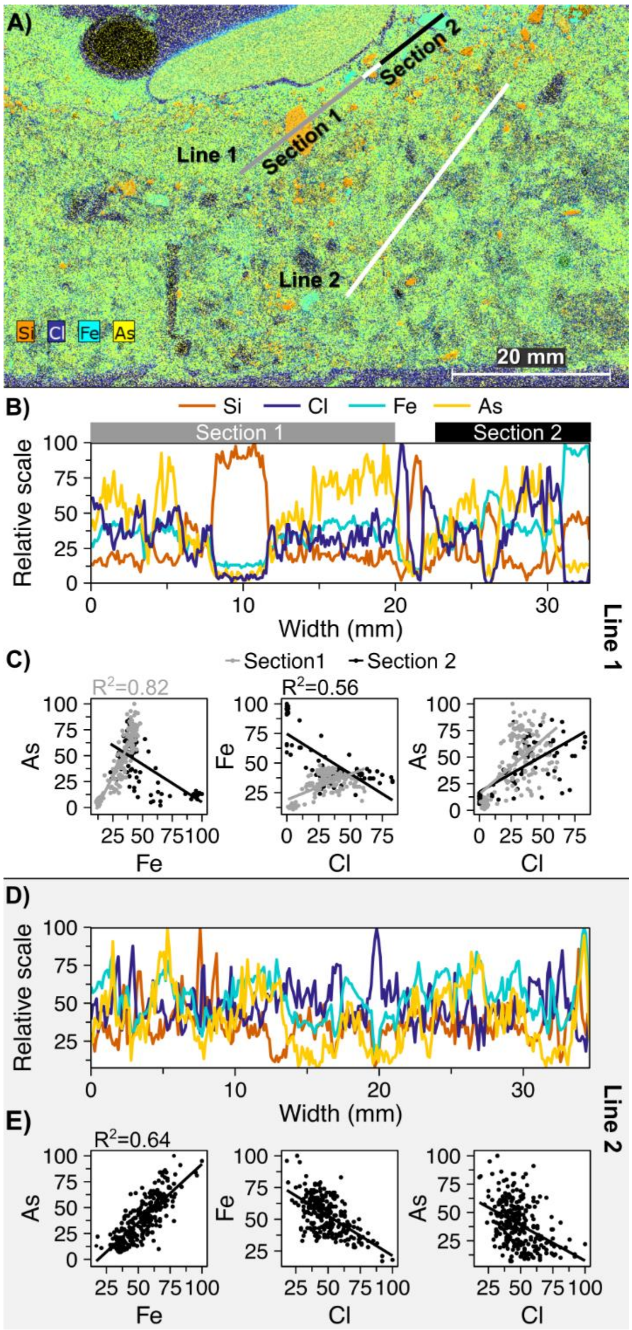

Figure S14. A) Superimposed µXRF image of element distributions in a slice of Bossegraben soil, showing arsenic (yellow), iron (blue), chlorine (dark purple), and silicon (dark orange) (pixel size 100 µm, scan time 5 ms/px) , as well as the location of diagonal profile lines 1 and 2. Chlorine is mapped in addition to the analytes of interest due to its high concentration in the resin, which allowed to differentiate between solids and pore space. Silicon is mapped as a proxy for the solid mineral structure of the soil. Separate µXRF images of each element can be found in Supp. Figs. S15-S18. B and D) Diagonal line scans showing relative counts intensities of Cl, As, and Fe along profile lines 1 and 2 (scan time 30 ms/px). In B, line 1 is divided into

two sections, differentiated as black and gray in C. C and E) correlation plots of relative counts of Cl, As, and Fe of profile lines 1 and 2. R^2 is shown only when above 0.5, $p<0.001$ for all correlations shown. All regression equations, p, and R^2 values can be found in Supp. Table S3.

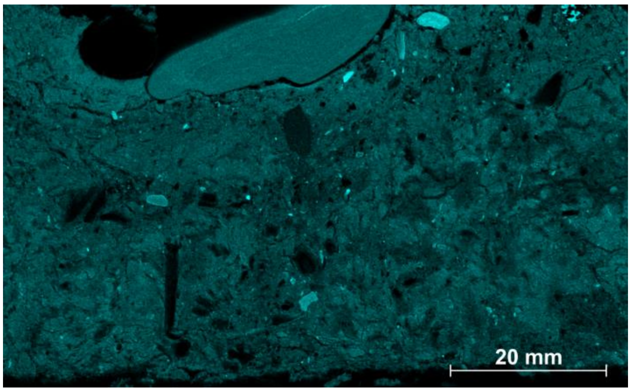

Figure S15. µXRF image of iron distribution (net intensity counts) in a slice of Bossegraben soil (Pixel size 100 µm, scan time 5 ms/px).

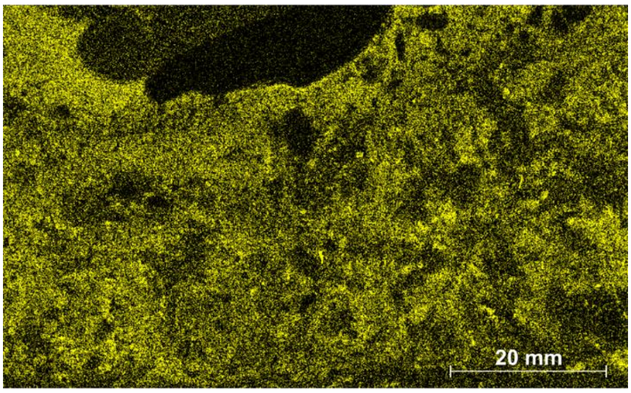

Figure S16. µXRF image of arsenic distribution (net intensity counts) in a slice of Bossegraben soil (Pixel size 100 µm, scan time 5 ms/px).

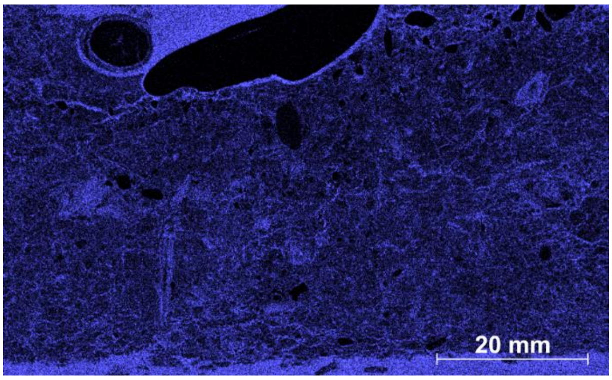

Figure S17. µXRF image of chlorine distribution (net intensity counts) in a slice of Bossegraben soil (Pixel size 100 µm, scan time 5 ms/px).

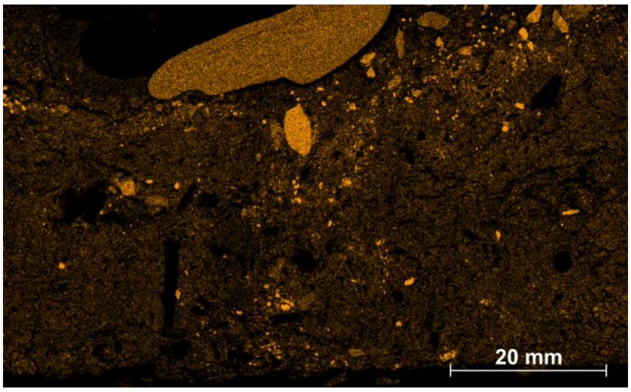

Figure S18. µXRF image of silicon distribution (net intensity counts) in a slice of Bossegraben soil (Pixel size 100 µm, scan time 5 ms/px).

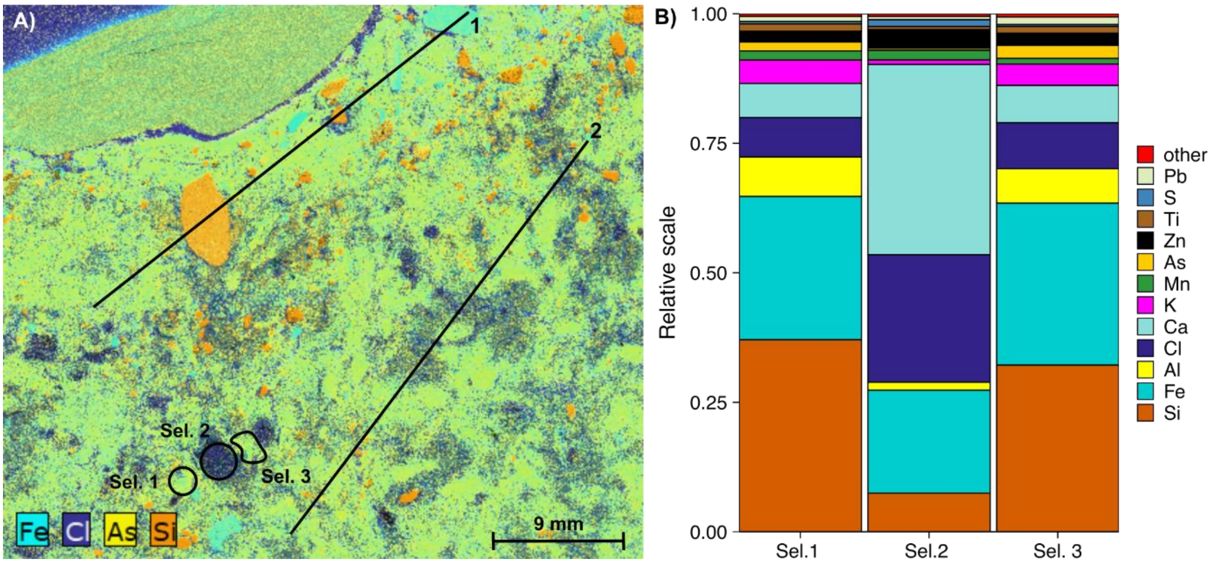

Figure S19. A) Overlay of µXRF scans of iron (blue), chlorine (dark purple), arsenic (yellow), and silicon (orange) with transect lines 1-2 (see Figure S14B-E for results), and selections 1-3 B) relative element composition extracted from selections 1-3 across a pore space. (Pixel size 100 µm, scan time 30 ms/ pixel)

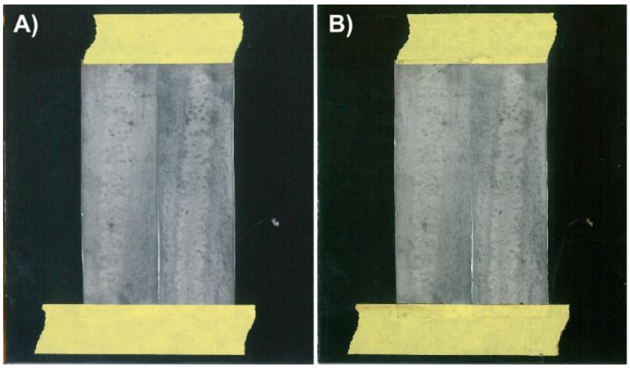

Figure S20. Scanned silver plates for the detection of sulfide, A) before deployment of the DET sampling probe, and B) after deployment. Upon exposure to sulfide, the formation of dark brown or black precipitate on the silver surface is expected.

4. Supplement to Tables

Supplementary Table S1. Total and percent recovery of As(V), As(III), and in iP mixed samples prepared in artificial saltwater. Calculations were based on the following: For both sets of equations, reagent H was measured at 1 hour of reaction time, the reagent L at 2:45 hours, and reagent LO at 2:45 hours of reaction time. <u>RGB ratio:</u> R_{640} divided by R_{460} for all reagents; <u>Multiple spectra:</u> RI_H) area under the curve between 700 nm and 715 nm, with a baseline from 485 nm to 776 nm, RI_L) area under the curve between 510 nm and 591 nm, with a baseline from 510 nm to 725 nm, RI_{LO}) area under the curve between 600 nm and 675 nm, with a baseline from 596 nm to 710 nm.

	[As(V)]= 10 µM, [iP]= 10 µM, [As(III)]=10 µM		[As(V)]= 5 µM, [iP]= 25 µM		[As(V)]= 25 µM, [iP]= 5 µM	
	Multiple Spectra	RGB ratio	Multiple Spectra	RGB ratio	Multiple Spectra	RGB ratio
As(V)	8.9 µM (89%)	4.5 µM (45%)	-0.2 µM (-4%)	-2 µM (-40%)	16.3 µM (65 %)	11.1 µM (44%)
iP	13.2 µM (132%)	16.1 µM (161%)	25.9 µM (104%)	30.9 µM (124%)	8.4 µM (169%)	8.4 µM (169%)
As(III)	12.0 µM (120%)	-53 µM (-530%)	-		-	

Supplementary Table S2. Regression equations and corresponding p and R^2 values for all correlations shown in Fig. S12-S14 and Fig. 5 in the main manuscript. R^2 over 0.5 are highlighted in bold.

		Equation	P	R^2
Figure S12B Box 1	iP vs As(V)	y=-0.72x+7.08	$1.0*10^{-131}$	**0.85**
	iP vs As(III)	y=0.05x+3.1	0.4	0
	iP vs Fe_{Tot}	y=0.08x+3.12	-	0
Figure S12B Box 2	iP vs As(V)	y=-0.83x+7.25	$2.2*10^{-106}$	**0.76**
	iP vs As(III)	y=0.08x+2.55	-	0
	iP vs Fe_{Tot}	y=0.08x+2.55	-	0
Figure S12B Box 3	iP vs As(V)	y=-0.83x+6.7	$5.6*10^{-116}$	**0.83**
	iP vs As(III)	y=0.08x+1.19	-	0
	iP vs Fe_{Tot}	y=0.08x+1.19	-	0
Figure S13A Box 1	iP vs As(V)	y=0x+0.01	0.11	0
	iP vs As(III)	y=0x+0.01	0.67	0
	iP vs Fe_{Tot}	y=0x+0.01	-	0
Figure S13A Box 2	iP vs As(V)	y=0.07x+1.25	$1.6*10^{-8}$	0.01
	iP vs As(III)	y=0.07x+1.37	$1.4*10^{-31}$	0.04
	iP vs Fe_{Tot}	y=0.03x+1.48	-	0
Figure S13A Box 3	iP vs As(V)	y=0x+0.04	0.23	0
	iP vs As(III)	y=-0.01x+0.03	0.40	0
	iP vs Fe_{Tot}	y=0x+0.03	0.65	0
Figure S13A Box 4	iP vs As(V)	y=0x+0.03	0.70	0
	iP vs As(III)	y=0.01x+0.02	$9.5*10^{-3}$	0
	iP vs Fe_{Tot}	y=0x+0.03	0.70	0
Figure S13A	iP vs As(V)	y=0x+0	0.57	0

Box 5	iP vs As(III)	y=0x+0	0.68	0
	iP vs Fe_{Tot}	y=0x+0	0.23	0
Figure S13A Box 6	iP vs As(V)	y=0x+0	-	-
	iP vs As(III)	y=0x+0	-	-
	iP vs Fe_{Tot}	y=0x+0	-	-
Figure S13A Box 7	iP vs As(V)	y=0x+0	0.44	0
	iP vs As(III)	y=0x+0	0.53	0
	iP vs Fe_{Tot}	y=0x+0	0.26	0
Figure S13A Box 8	iP vs As(V)	y=0x+0	0.92	0
	iP vs As(III)	y=0x+0	0.30	0
	iP vs Fe_{Tot}	y=0x+0	0.83	0
Figure S13A Box 9	iP vs As(V)	y=0x+0.01	0.89	0
	iP vs As(III)	y=0x+0.01	0.36	0
	iP vs Fe_{Tot}	y=0x+0.01	0.68	0
Figure S13A Box 10	iP vs As(V)	y=0x+0	0.50	0
	iP vs As(III)	y=0x+0	0.30	0
	iP vs Fe_{Tot}	y=0x+0	0.88	0
Figure S13A Box 11	iP vs As(V)	y=0x+0	-	-
	iP vs As(III)	y=0x+0	-	-
	iP vs Fe_{Tot}	y=0x+0	-	-
Figure S13A Box 12	iP vs As(V)	y=0x+0	0.87	0
	iP vs As(III)	y=0x+0	0.33	0
	iP vs Fe_{Tot}	y=0x+0	0.99	0
Figure S13A	iP vs As(V)	y=0x+0	-	-

Box 13	iP vs As(III)	y=0x+0	-	-
	iP vs Fe_{Tot}	y=0x+0	-	-
Figure S13A Box 14	iP vs As(V)	y=0x+1	-	-
	iP vs As(III)	y=0x+2	-	-
	iP vs Fe_{Tot}	y=0x+3	-	-
Figure S14C Line 1 Complete	As vs Fe	y=0.08x+40.98	0.39	0.00
	Fe vs Cl	y=-0.14x+42.95	$2.5*10^{-2}$	0.02
	As vs Cl	y=0.81x+18.77	$7.1*10^{-24}$	0.35
Figure S14C Line 1 Section 1	As vs Fe	y=2.28x-26.77	$2.9*10^{-61}$	**0.82**
	Fe vs Cl	y=0.43x+18.94	$2.1*10^{-18}$	0.39
	As vs Cl	y=1.03x+15.26	$3.6*10^{-16}$	0.35
Figure S14C Line 1 Section 2	As vs Fe	y=-0.73x+78.14	$8.3*10^{-12}$	0.45
	Fe vs Cl	y=-0.68x+74.69	$1.1*10^{-15}$	**0.56**
	As vs Cl	y=0.69x+17.34	$1.2*10^{-12}$	0.47
Figure S14E Line 2	As vs Fe	y=1.13x-20.89	$2.3*10^{-62}$	**0.64**
	Fe vs Cl	y=-0.63x+84.23	$1.8*10^{-28}$	0.36
	As vs Cl	y=-0.63x+70.08	$1.9*10^{-13}$	0.18
Figure 5A	Fe_{Tot} vs As(V)	y=19.17x+239.42	$6.8*10^{-49}$	0.11
	Fe_{Tot} vs As(III)	y=9.52x+134.82	$3.6*10^{-191}$	0.37
	As(V) vs As(III)	y=0.12x+0.19	$8.2*10^{-89}$	0.19
Figure 5B	Fe_{Tot} vs As(V)	y=6.08x+252.69	$3.6*10^{-3}$	0.18
	Fe_{Tot} vs As(III)	y=-1.84x+335.66	$9.4*10^{-2}$	0.06
	As(V) vs As(III)	y=-0.42x+16.76	$1.8*10^{-12}$	**0.69**
Figure 5C	Fe_{Tot} vs As(V)	y=38.14x+31.25	$1.0*10^{-5}$	0.37

	Fe_{Tot} vs As(III)	y=-27.45x+863.31	$1.8*10^{-8}$	**0.52**
	As(V) vs As(III)	y=-0.17x+11.14	$5.7*10^{-2}$	0.08
Figure 5D	Fe_{Tot} vs As(V)	y=14.31x+381.53	$5.3*10^{-2}$	0.08
	Fe_{Tot} vs As(III)	y=17.39x+66.92	$8.3*10^{-16}$	**0.78**
	As(V) vs As(III)	y=0.08x+6.68	0.19	0.04

5. Supplement to References

(1) Robertson, D.; Teasdale, P. R.; Welsh, D. T. A Novel Gel-Based Technique for the High Resolution, Two-Dimensional Determination of Iron (II) and Sulfide in Sediment. *Limnol. Oceanogr. Methods* **2008**, *6* (10), 502–512. https://doi.org/10.4319/lom.2008.6.502.

(2) Cesbron, F.; Metzger, E.; Launeau, P.; Deflandre, B.; Delgard, M.-L.; Thibault de Chanvalon, A.; Geslin, E.; Anschutz, P.; Jézéquel, D. Simultaneous 2D Imaging of Dissolved Iron and Reactive Phosphorus in Sediment Porewaters by Thin-Film and Hyperspectral Methods. *Environ. Sci. Technol.* **2014**, *48* (5), 2816–2826. https://doi.org/10.1021/es404724r.

(3) O'Reilly, S. E.; Strawn, D. G.; Sparks, D. L. Residence Time Effects on Arsenate Adsorption/Desorption Mechanisms on Goethite. *Soil Sci. Soc. Am. J.* **2001**, *65* (1), 67–77. https://doi.org/10.2136/sssaj2001.65167x.
